# Supplementary material for: De novo birth of functional microproteins in the human lineage
Source: Cell Rep. 2022 Dec 20;41(12):111808. doi: 10.1016/j.celrep.2022.111808 (PMC10073203; doi:10.1016/j.celrep.2022.111808)
Supplement: Document S1. Figures S1–S7 [file mmc1.pdf]

Cell Reports, Volume *41*

Supplemental information

De novo birth of functional microproteins in the human lineage

Nikolaos Vakirlis, Zoe Vance, Kate M. Duggan, and Aoife McLysaght

Supplementary Figures

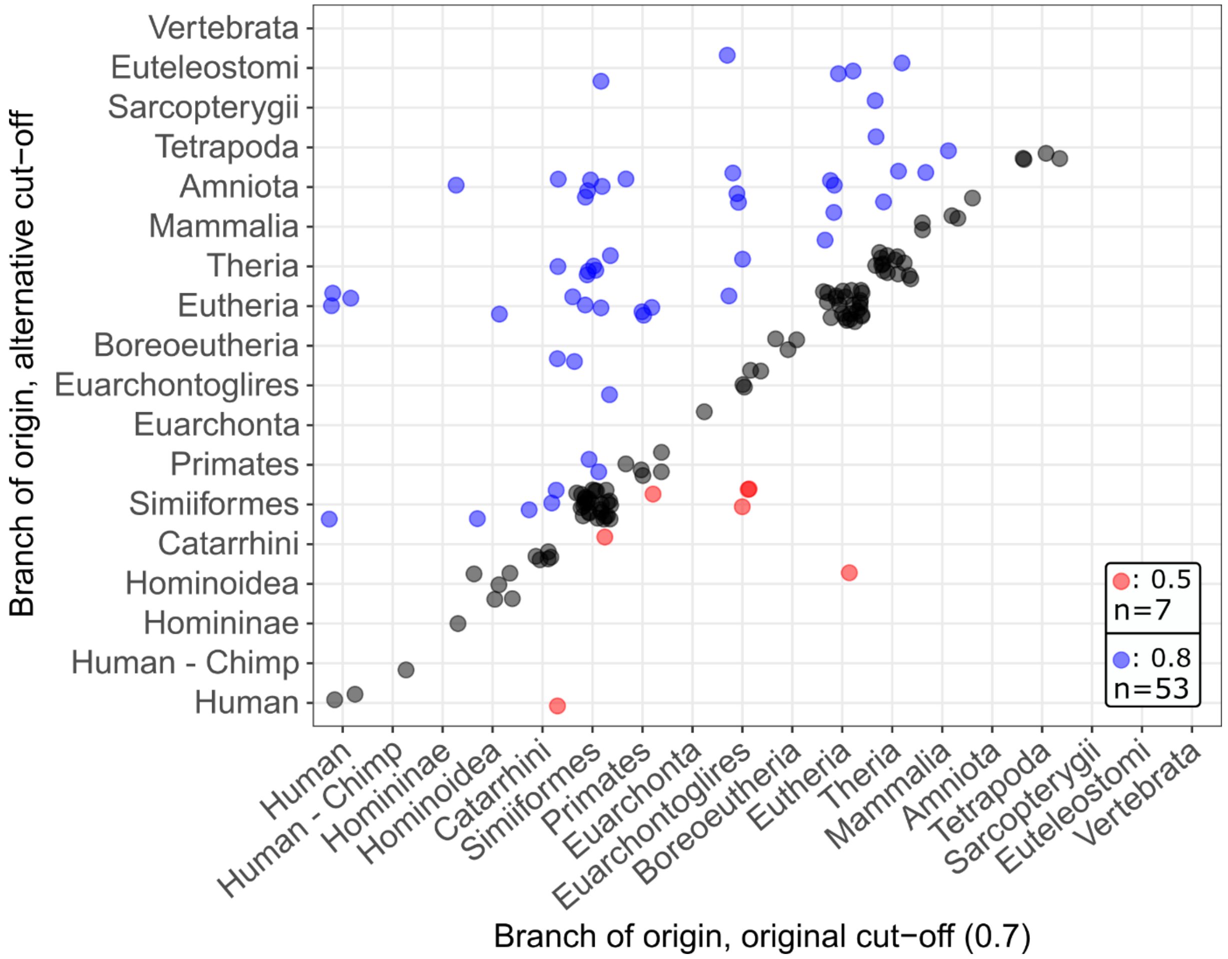

Figure S1: **Alternative intact length proportion cut-off comparison**. Effects of applying two alternative intact length proportion cut-offs, one stricter (red points) and one more relaxed (blue points) on predicted branch of ORF origin for 155 de novo originated ORFs. Related to Figure 1.

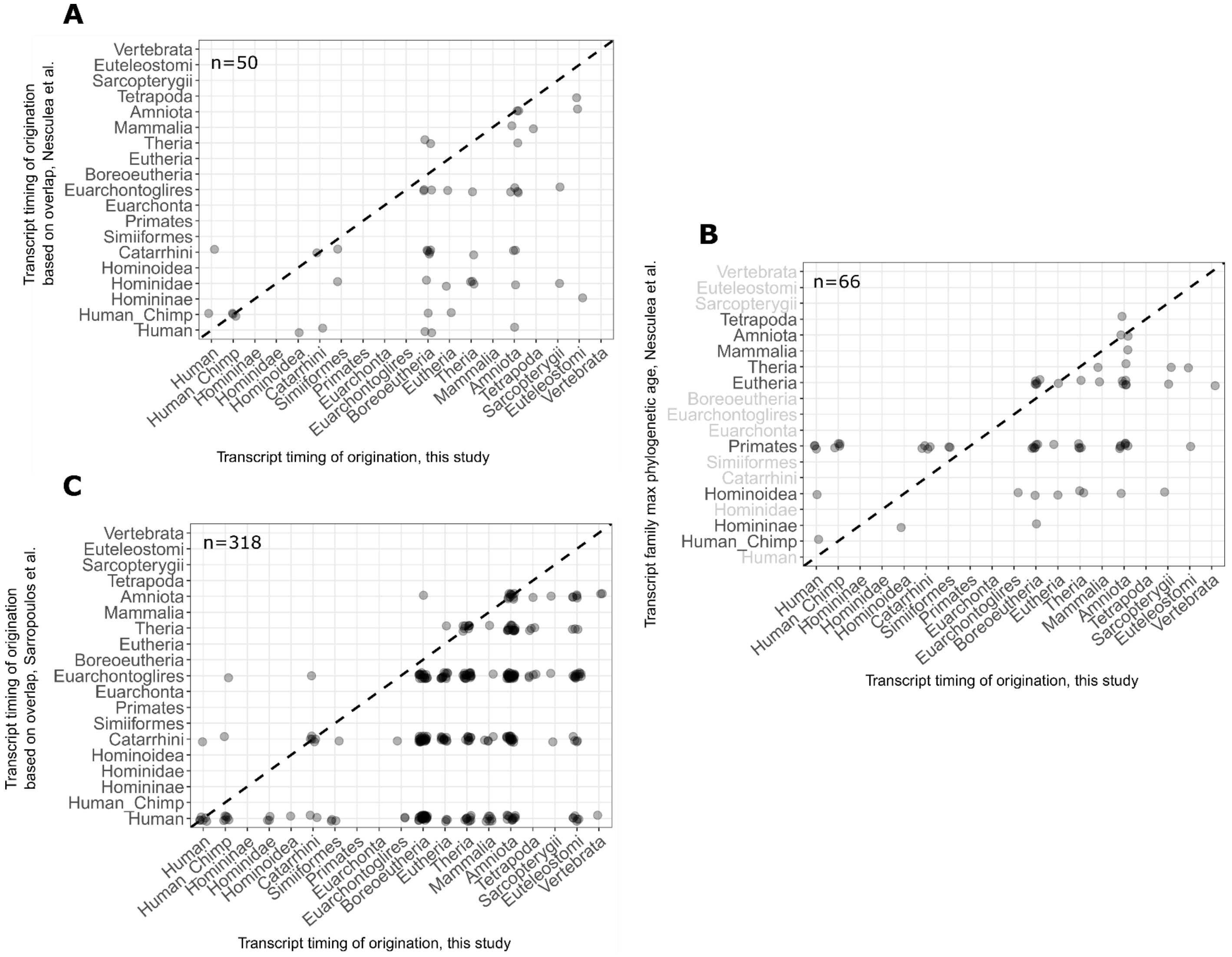

Figure S2: Transcript timing of origination comparison. A: Transcript timing of origination comparison using our data and using the assembled transcriptome data from Necsulea et al., relying on overlap of exonic regions and calculated by us (40/50 ages are older using our data, 4/50 ages are older using Nesculea et al. data, 6/50 the ages are the same; avg. time differential is 107.1my) B: Same comparison but using overlap of entire transcript coordinates of Necsulea et al. and taking the transcript family maximum age as calculated by Necsulea et al (numbers as in A, 42/66, 21/66, 3/66; time differential is 87.76my) C: In the same manner as in A, but using the data from Sarropoulos et al. (numbers as in A: 283/318, 6/318, 29/318; time differential is 134.8my) Related to Figure 1.

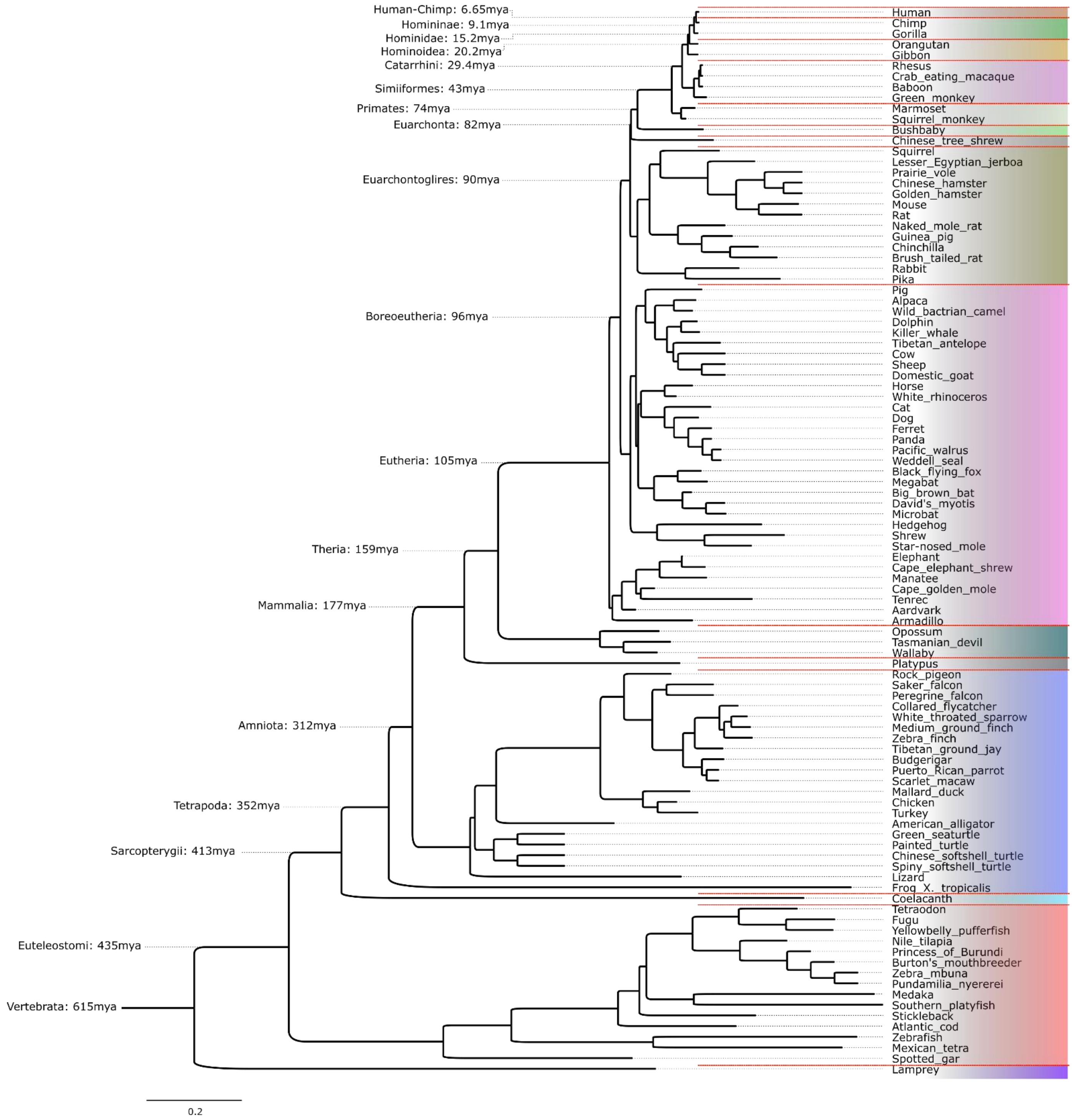

Figure S3: Phylogeny of species included in this study. UCSC Genome Browser 100-way phylogenetic tree with common species names, visualized using FigTree (https://github.com/rambaut/figtree) and annotated by us with the human ancestral branches and their ages as estimated by TimeTree (http://www.timetree.org/). Colors and red lines help distinguish the species descending from each ancestral human branch. Related to Figure 1.

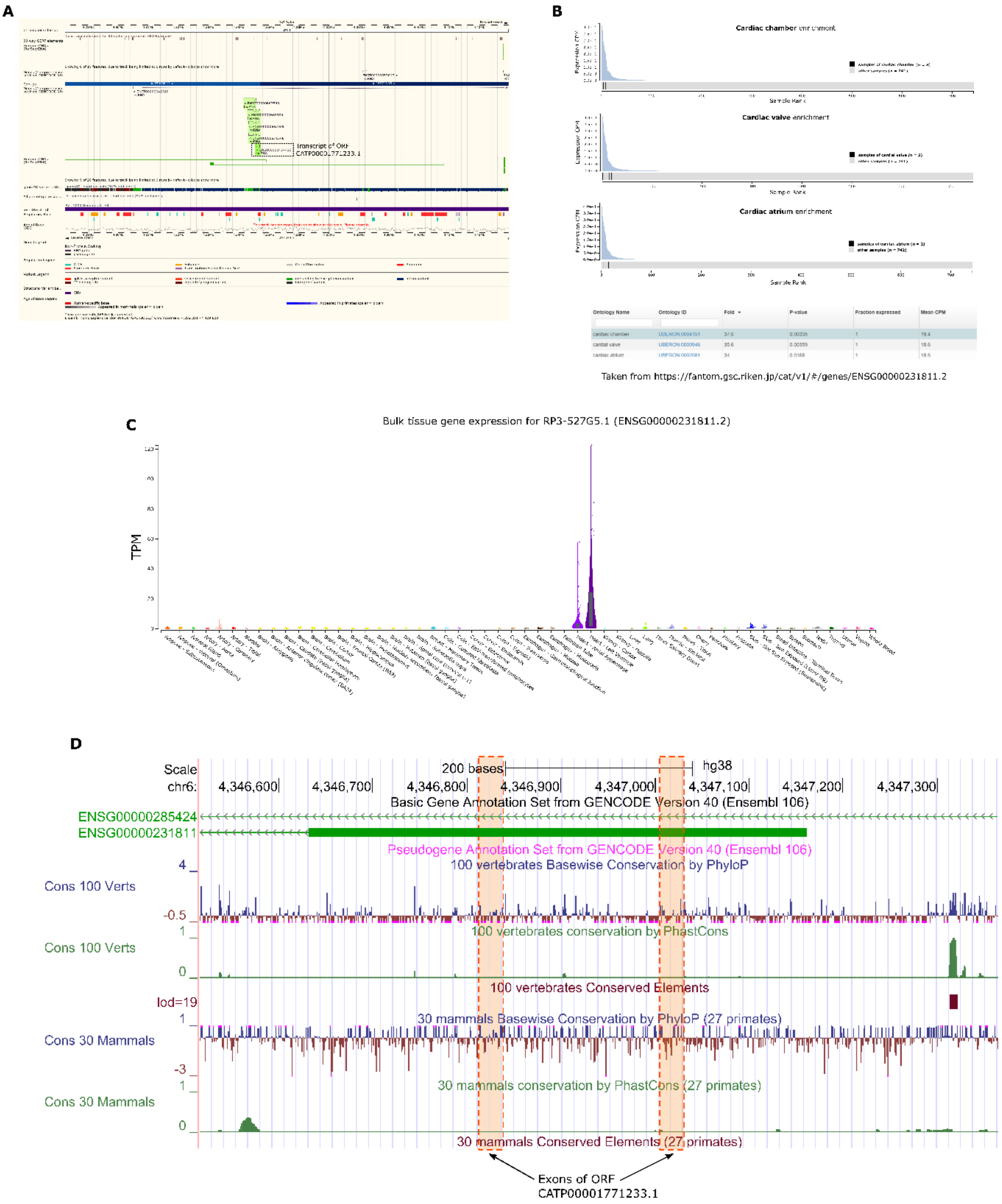

Figure S4: Genomic, expression and conservation data for ENSG00000231811.2. A: Genome browser view from ENSEMBL of the region of gene ENSG00000231811.2. The transcripts of the gene are highlighted in green. B: Sample ontology enrichment (three highest ranking ones) detected by Hon et al. for gene ENSG00000231811.2. Plots show the ranks of the samples for each ontology, table shows the relevant information. Taken from https://fantom.gsc.riken.jp/cat/v1/#/genes/ENSG00000231811.2 C: Expression plot taken from GTeX https://gtexportal.org/home/gene/RP3-527G5.1#geneExpression . D: UCSC

Genome Browser view of the region of the exemplar ORF; only conservation tracks are shown. Related to Figure 2.

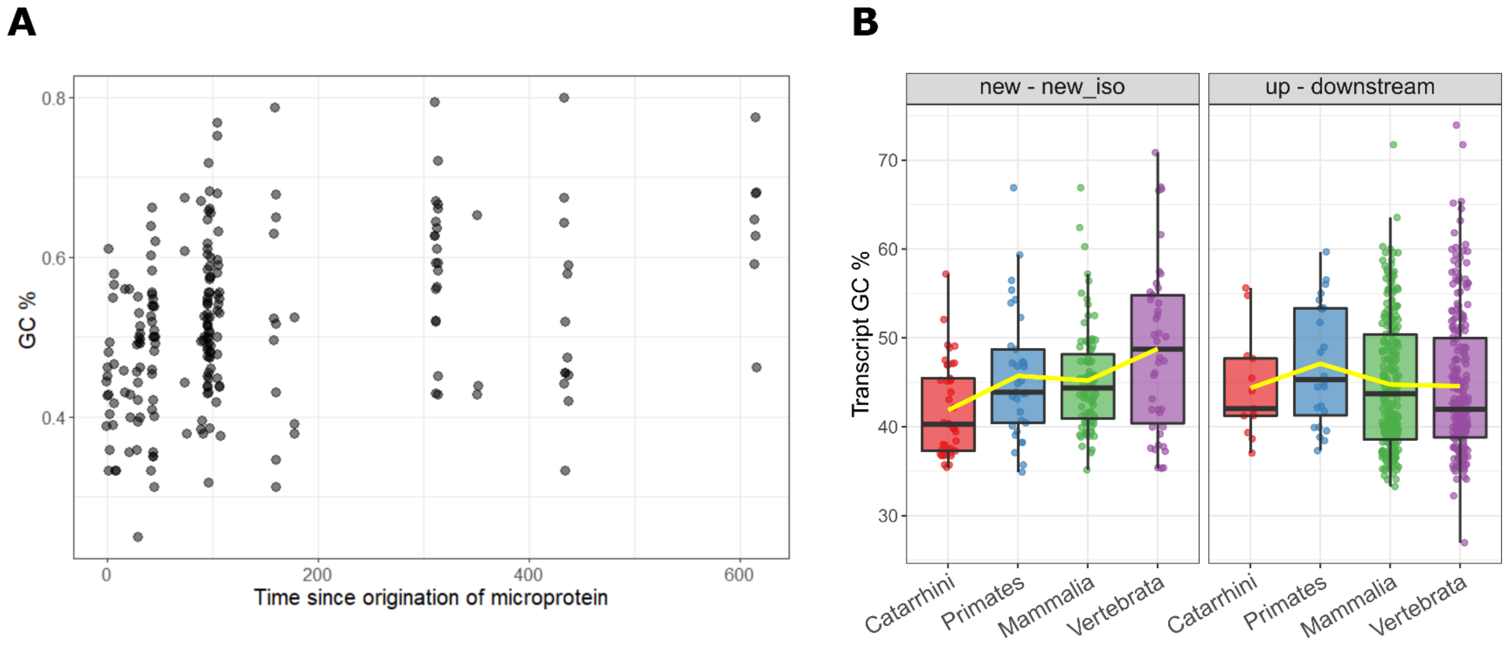

Figure S5: ORF and transcript GC% and timing of origination. A: Correlation of ORF GC% to time since putative origin of microprotein, for ORFs on new/new_iso transcripts (Spearman's Rho=0.36, P-value=$5x10^{-8}$). B: GC% of main transcripts for ORFs in the four broad groups of origin. Difference between Catarrhini and Vertebrata groups in the "new – new_iso" type is statistically significant (Wilcoxon test P-value=0.0004, means of 41.9 vs. 48.8). Contrary to ORF GC%, there is no statistically significant difference between those in the "new – new_iso" and those in the "up – downstream" types (P-value = 0.16). Related to Figure 3.

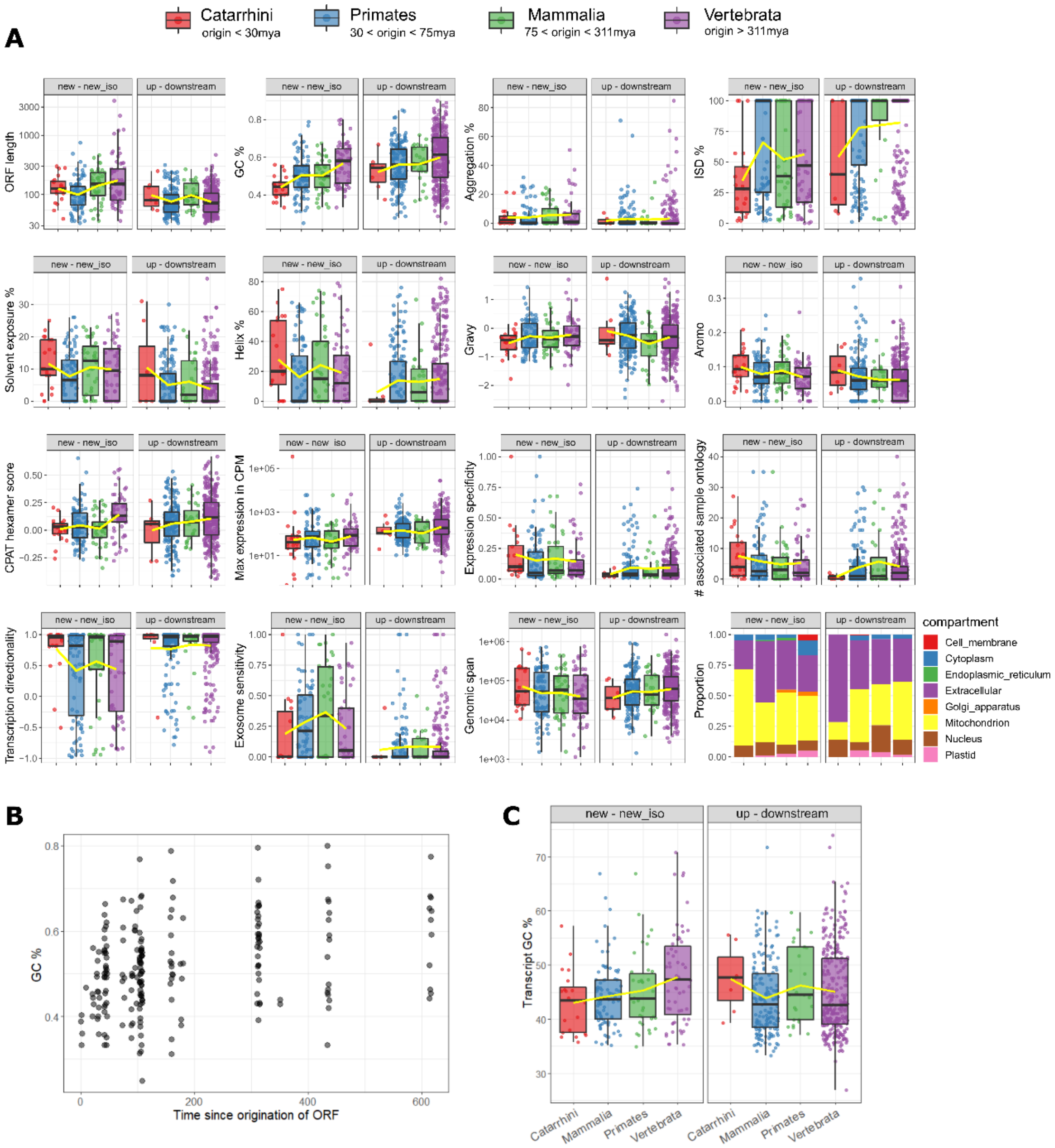

Figure S6: Distributions of various ORF, transcript and protein properties (ORF-based timing of origination definition). A: Distributions of various ORF, transcript and protein properties for all 715 microproteins, in four broad groups of putative origin age, defined using ORF timing or origination only, for all cases. Yellow line connects the averages across the groups. B: Correlation of ORF GC% to timing of origination of ORF, for ORFs on new/new_iso transcripts (Spearman's Rho=0.32, P-value=1.8×10^{-6}). C: GC% of main transcripts for ORFs in the four broad groups of origin, defined by ORF origin. Difference between Catarrhini and Vertebrata groups in the "new – new_iso" type is statistically significant (Wilcoxon test P-value=0.025, means of 43.1 vs. 47.8). Related to Figure 3.

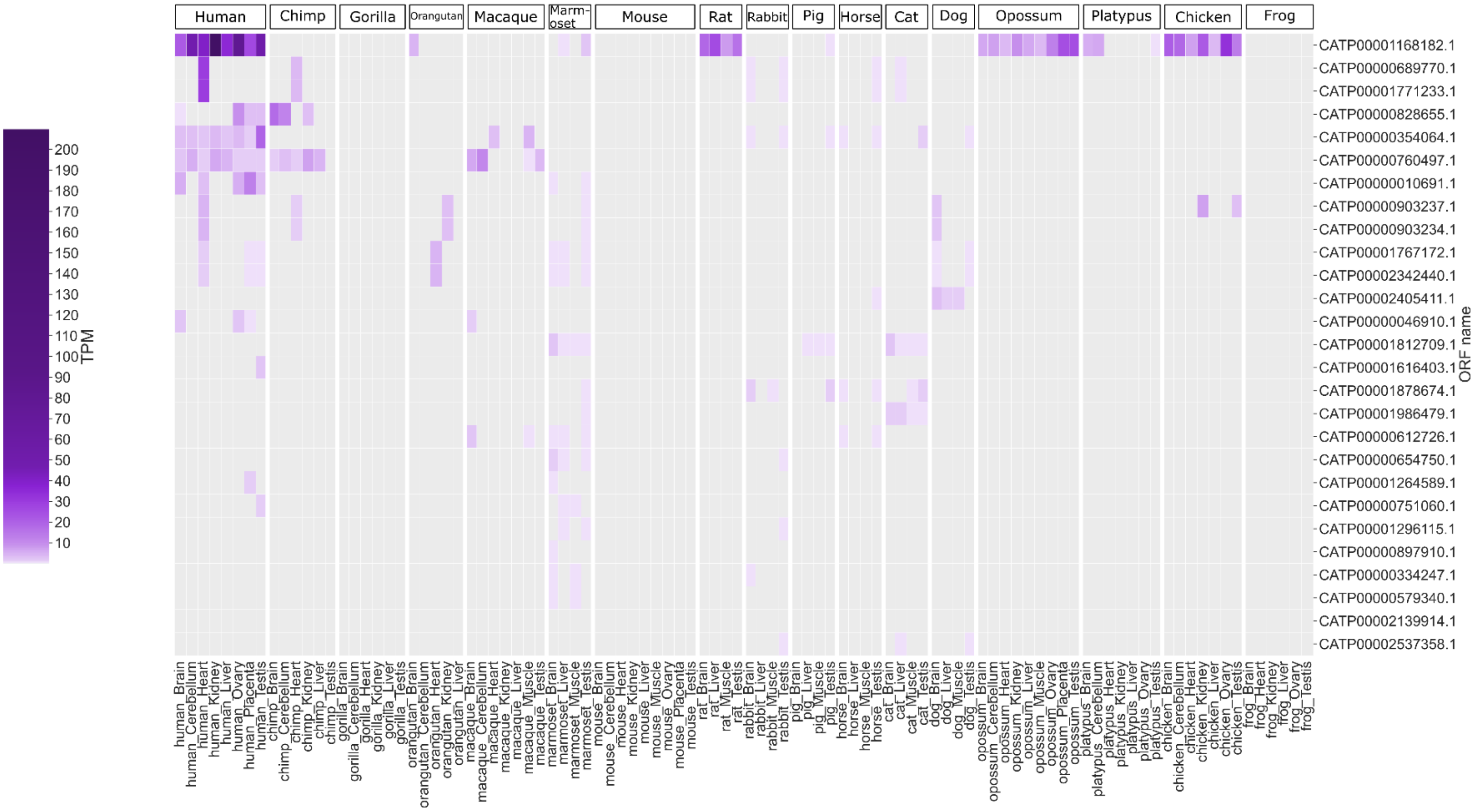

Figure S7: Expression levels of 27 ORFs initially predicted to be human-specific. Heatmap showing expression levels in TPM for each of the 27 ORFs initially predicted to be human-specific (based on analysis of reference transcriptomes) in the different tissue samples analyzed. 9/27 remain human-specific after filtering and integration of these data (see Methods). Related to Methods.
