# Supplementary material for: De novo birth of functional microproteins in the human lineage
Source: Cell Rep. 2022 Dec 20;41(12):111808. doi: 10.1016/j.celrep.2022.111808 (PMC10073203; doi:10.1016/j.celrep.2022.111808)
Supplement: Table S5. Species, tissues, and SRA accessions for RNA-seq datasets used to infer transcription of orthologous loci, related to STAR Methods and Figure S6 [file mmc6.docx]

**Supplementary Table 5**

| Species | Tissues | SRA accessions | References |
| --- | --- | --- | --- |
| Human | Brain, Cerebellum, Heart, Kidney, Liver, Ovary, Placenta, Testis | ERR2812349, ERR2812350, ERR2812351, SRR306844, SRR306845, SRR306846, SRR306847, SRR306848, SRR306849, SRR306850, SRR306851, SRR306852, SRR306853, ERR2812355, ERR2812356, ERR2812357, SRR649364, SRR943341,  SRR649363, SRR943340, SRR943354, SRR943359, ERR2812361, ERR2812362, ERR2812363 | **Wang 2020 Nature**  **Brawand 2011 Nature**  **Nesculea et al. 2014 Nature** |
| Chimp | Brain, Cerebellum, Heart, Kidney, Liver, Testis | SRR306811, SRR306812, SRR306813, SRR306814, SRR306815, SRR306816,  SRR306817, SRR306818,  SRR306819, SRR306820,  SRR306821, SRR306822,  SRR306823, SRR306824,  SRR306825 | **Brawand 2011 Nature** |
| Gorilla | Brain, Cerebellum, Heart, Kidney, Liver, Testis | SRR306800, SRR306801,  SRR306802, SRR306803, SRR306804, SRR306805, SRR306806, SRR306807,  SRR306808, SRR306809,  SRR306810 | **Brawand 2011 Nature** |
| Orangutan | Brain, Cerebellum, Heart, Kidney, Liver | SRR306791, SRR306792,  SRR306793, SRR306794, SRR306795, SRR306796, SRR306797, SRR306798, SRR306799 | **Brawand 2011 Nature** |
| Macaque | Brain, Cerebellum, Heart, Kidney, Liver, Muscle, Testis | ERR2812367, ERR2812368, ERR2812369, SRR306780, SRR306781, SRR306782, SRR306783, SRR306784, SRR306785, ERR2812373, ERR2812374,ERR2812375,  ERR3417964, ERR3417996, ERR3418000, ERR2812379, ERR2812380, ERR2812381 | **Wang 2020 Nature**  **Brawand 2011 Nature**  **Roller 2021 Genome Biology** |
| Marmoset | Brain, Liver, Muscle, Testis | ERR3417915, ERR3417984, ERR3417986, ERR3417938, ERR3417970, ERR3417975, ERR3417914, ERR3417968, ERR3417987, ERR3417930, ERR3417944, ERR3417962 | **Roller 2021 Genome Biology** |
| Mouse | Brain, Cerebellum, Heart, Kidney, Liver, Muscle, Ovary, Placenta, Testis | ERR2812385, ERR2812386, ERR2812387, SRR306763, SRR306764, SRR306765, SRR306766, SRR306767, SRR306768, SRR306769, SRR306770, SRR306771,  ERR2812391, ERR2812392, ERR2812393, ERR2812442, ERR2812443, ERR3417912, ERR3417983, ERR3418004, SRR649372, SRR943342, SRR943343, SRR649373, SRR649374, SRR943344, SRR943345, SRR943355, SRR943356, ERR2812397, ERR2812398, ERR2812399 | **Wang 2020 Nature**  **Brawand 2011 Nature**  **Nesculea et al. 2014 Nature**  **Roller 2021 Genome Biology** |
| Rat | Brain, Liver, Muscle, Testis | ERR3417910, ERR3417913, ERR3417949, ERR3417917, ERR3417992, ERR3418010,  ERR3417901, ERR3417903, ERR3417991, ERR3417900, ERR3417906, ERR3417950 | **Roller 2021 Genome Biology** |
| Rabbit | Brain, Liver, Muscle, Testis | ERR3417909, ERR3417957, ERR3417997, ERR3417919, ERR3417945, ERR3417948,  ERR3417959, ERR3417978, ERR3417982, ERR3417971, ERR3417973,ERR3417980 | **Roller 2021 Genome Biology** |
| Pig | Brain, Liver, Muscle, Testis | ERR3417916, ERR3418014, ERR3418018, ERR3417918, ERR3417924, ERR3417937,  ERR3417935, ERR3417965, ERR3417993, ERR3417904, ERR3417952, ERR3418012 | **Roller 2021 Genome Biology** |
| Horse | Brain, Liver, Muscle, Testis | ERR3418005, ERR3418007, ERR3418011, ERR3417940, ERR3417956, ERR3417976,  ERR3417966, ERR3417981, ERR3417988, ERR3417911, ERR3417951, ERR3417969 | **Roller 2021 Genome Biology** |
| Cat | Brain, Liver, Muscle, Testis | ERR3417907, ERR3417925, ERR3417967, ERR3417927, ERR3417928, ERR3417934,  ERR3417923, ERR3417932, ERR3417979, ERR3417926, ERR3417931, ERR3417933 | **Roller 2021 Genome Biology** |
| Dog | Brain, Liver, Muscle, Testis | ERR3417943, ERR3417972, ERR3417974, ERR3417942, ERR3417961, ERR3417977,  ERR3417929, ERR3417936, ERR3417999, ERR3417958, ERR3417960, ERR3417985 | **Roller 2021 Genome Biology** |
| Opossum | Brain, Cerebellum, Heart, Kidney, Liver, Muscle, Ovary, Placenta, Testis | ERR2812403, ERR2812404, ERR2812405, SRR306745, SRR306746, SRR306747, SRR306748, SRR306749, SRR306750, SRR306751, SRR306752, ERR2812409, ERR2812410, ERR2812411, ERR3417939, ERR3417954, ERR3417989, SRR649377, SRR943346, SRR649378, SRR943347, SRR943348,  ERR2812415, ERR2812416, ERR2812417 | **Wang 2020 Nature**  **Brawand 2011 Nature**  **Nesculea et al. 2014 Nature**  **Roller 2021 Genome Biology** |
| Platypus | Brain, Cerebellum, Heart, Kidney, Liver, Ovary, Testis | ERR2812421, ERR2812422, ERR2812423, SRR306728, SRR306729, SRR306730, SRR306731, SRR306732, SRR306733, SRR306734,  ERR2812427, ERR2812428, ERR2812429, SRR649382, SRR943349, SRR943350,  ERR2812433, ERR2812434, ERR2812435 | **Wang 2020 Nature**  **Brawand 2011 Nature**  **Nesculea et al. 2014 Nature** |
| Chicken | Brain, Cerebellum, Heart, Kidney, Liver, Ovary, Testis | ERR2812331, ERR2812332, ERR2812333, SRR306712, SRR306713, SRR306714, SRR306715, SRR306716, SRR306717, ERR2812337, ERR2812338, ERR2812339, ERR2812438, ERR2812439,  SRR649386, SRR649387, SRR649388, SRR943351,  ERR2812343, ERR2812344, ERR2812345 | **Wang 2020 Nature**  **Brawand 2011 Nature**  **Nesculea et al. 2014 Nature** |
| Frog | Brain, Heart, Kidney, Liver, Ovary, Testis | SRR649391, SRR649392,  SRR649393, SRR649394,  SRR649395, SRR649396,  SRR649397, SRR649398,  SRR649400, SRR943352,  SRR649399, SRR943353 | **Nesculea et al. 2014 Nature** |
