# Supplementary material for: De novo birth of functional microproteins in the human lineage
Source: Cell Rep. 2022 Dec 20;41(12):111808. doi: 10.1016/j.celrep.2022.111808 (PMC10073203; doi:10.1016/j.celrep.2022.111808)
Supplement: Document S2. Article plus supplemental information [file mmc8.pdf]

Cell Reports

Article

De novo birth of functional microproteins in the human lineage

Graphical abstract

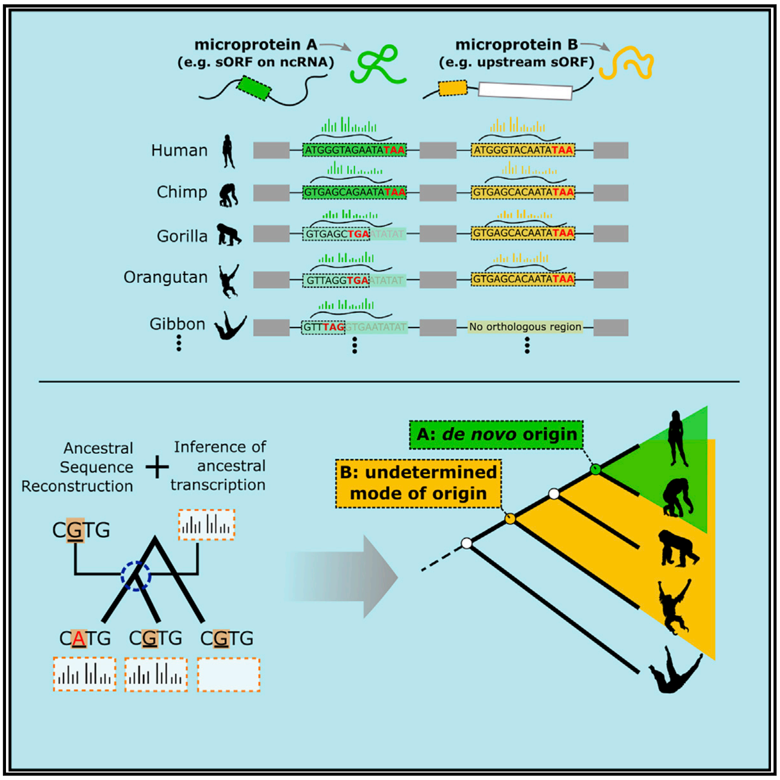

Authors

Nikolaos Vakirlis, Zoe Vance, Kate M. Duggan, Aoife McLysaght

Correspondence

vakirlisnikos@gmail.com (N.V.),
aoife.mclysaght@tcd.ie (A.McL.)

In brief

Human microproteins encoded by small ORFs have been found to be functional. By comparing the corresponding sequences across vertebrate genomes, Vakirlis et al. show that a number of these originated "from scratch" from noncoding sequences, including two very recent cases unique to humans. These cases demonstrate the rapid evolution of genetic novelty.

Highlights

- We estimate the evolutionary origins of functional human microproteins
- Some are novel, having originated entirely *de novo* from noncoding sequences
- These mostly lack sequence signals of conservation and selection
- Many more novel ones could exist and escape detection

Vakirlis et al., 2022, Cell Reports *41*, 111808
December 20, 2022 © 2022 The Author(s).
https://doi.org/10.1016/j.celrep.2022.111808

CellPress

Cell Reports

CellPress
OPEN ACCESS

Article

De novo birth of functional microproteins in the human lineage

Nikolaos Vakirlis,[1,3,4,*] Zoe Vance,[2,3] Kate M. Duggan,[2] and Aoife McLysaght[2,*]
[1]Institute for Fundamental Biomedical Research, Biomedical Sciences Research Center "Alexander Fleming", Vari, Greece
[2]Smurfit Institute of Genetics, Trinity College Dublin, University of Dublin, Dublin, Ireland
[3]These authors contributed equally
[4]Lead contact
*Correspondence: vakirlisnikos@gmail.com (N.V.), aoife.mclysaght@tcd.ie (A.McL.)
https://doi.org/10.1016/j.celrep.2022.111808

SUMMARY

Small open reading frames (sORFs) can encode functional "microproteins" that perform crucial biological tasks. However, their size makes them less amenable to genomic analysis, and their origins and conservation are poorly understood. Given their short length, it is plausible that some of these functional microproteins have recently originated entirely *de novo* from noncoding sequences. Here we sought to identify such cases in the human lineage by reconstructing the evolutionary origins of human microproteins previously found to have measurable, statistically significant fitness effects. By tracing the formation of each ORF and its transcriptional activation, we show that novel microproteins with significant phenotypic effects have emerged *de novo* throughout animal evolution, including two after the human-chimpanzee split. Notably, traditional methods for assessing coding potential would miss most of these cases. This evidence demonstrates that the functional potential intrinsic to sORFs can be relatively rapidly and frequently realized through *de novo* gene emergence.

INTRODUCTION

It is now a well-established biological fact that many more open reading frames (ORFs) are translated than those traditionally annotated as protein coding.[1] Most of these so called "noncanonical" ORFs, such as ones found on long noncoding RNAs, are small, typically <300 nucleotides. While most of these are plausibly just biological noise, many encode functional microproteins.[2] Microproteins perform diverse functions through various mechanisms: some, encoded by upstream ORFs (uORFs), exert translational control over the main ORF of the transcript,[3] while others interact with larger protein complexes or with cellular membranes.[4,5] Microproteins have long been overlooked in genomic studies, mostly due to technical limitations linked to their small size.[6] But there is now increasing interest and investment toward identifying them and understanding their functions and possible roles in health and disease.[7,8] Well-studied examples of functional human microproteins include NoBody,[9] PIGBOS,[10] and myoregulin,[11] while many more have been identified in other species such as mouse,[12] plants,[13] bacteria,[14] and elsewhere.[15] Microproteins have been observed to be highly conserved over long evolutionary times in animals and in plants,[16–18] but they can also be evolutionarily novel.[5,19]

Evolutionarily novel genes can evolve out of preexisting ones through sequence divergence[20–22] (preceded by duplication or not), but they can also emerge entirely *de novo*, out of ancestrally non-genic genomic regions.[23] The process of *de novo* gene birth, as the latter is called, has now been studied extensively in multiple species such as yeast,[24–26] mouse,[27] flies,[28] fish,[29,30] rice[31] nematodes,[32] and human.[33] In humans, early studies relying on gene annotations[33–36] established that *de novo* genes can indeed form, even in as short an evolutionary time frame as the split of human from chimpanzee. Later studies adopted broader search strategies, starting from entire transcriptomes and incorporating ribosome profiling data to identify translated ORFs.[37,38]

While many studies have addressed the conservation of human microproteins, their modes of origin, *de novo* or otherwise, have not been systematically investigated. Indeed, conservation is widely used as a coding/functional signature, hence, non-conserved, novel ORFs are excluded from most studies. However, it is practically inevitable that novel genes will initially arise as ORFs coding for very small proteins[31,39] (with additional tendencies stemming from genomic mutational biases[40]). Given the fact that *de novo* gene birth seems to consistently result in short ORF sequences[23,41] (at least initially), and that microproteins perform functions out of simple structures, it follows that human microproteins could have recently emerged *de novo* and already assumed selectively relevant cellular functions. Thus, the study of microproteins and the study of *de novo* emerged genes naturally intersect.

Here, we leveraged the depth of a recently published dataset of human microproteins translated from noncanonical ORFs[42] to look for such evolutionary birth events. More specifically, we sought to identify and examine cases in the human lineage of small proteins that evolved out of previously noncoding

This is an open access article under the CC BY license (http://creativecommons.org/licenses/by/4.0/).

sequences and acquired function either immediately or shortly thereafter. This is doubly important: for our understanding of the intriguing, and still largely mysterious phenomenon of *de novo* gene birth, but also for our appreciation of the full functional potential of the human genome.

RESULTS

The reconstructed evolutionary origins of human microproteins

A recent rigorous analysis of ribosome profiling data by Chen et al. enabled ORF translation to be inferred with high confidence for hundreds of human noncanonical ORFs.[42] We used these data and focused on ORFs that did not overlap canonical, coding ones, i.e., are either located on transcripts previously annotated as noncoding ("new"); or upstream/downstream of known coding ORFs ("upstream" and "downstream" respectively); or new mRNA isoforms of an annotated protein-coding gene, but where the new isoform lacked an annotated coding ORF ("new_iso"), labeled per classification of Chen et al. (see STAR Methods). Furthermore, we only kept those ORFs that we could unambiguously match to ORFs identified and analyzed by Hon et al.[43] in their comprehensive human transcriptome atlas with accurate 5′ ends (FANTOM CAT). A total of 715 ORFs were included in the final dataset (499 "upstream," 179 "new," 32 "new_iso," and 5 "downstream"). They range from 33 to 3,825 nt in length, with a median of 81 nt.

For each ORF, we sought to estimate its evolutionary timing of origination (i.e., when the sequence of the ORF first formed) and to establish whether its evolutionary mode of origination was *de novo*. We searched for the orthologous chromosomal region of each human ORF in the genomes of 99 other vertebrate species (see STAR Methods, Table S1). For each ORF, the orthologous nucleotide sequences were aligned, and we constructed a phylogenetic tree following the species tree topology to estimate the branch lengths. Finally, ancestral sequence reconstruction (ASR) was performed, and the presence or not of an ORF at each human ancestral node was inferred. To decide whether an ORF was present or not, we applied a length ratio cutoff (length of ancestral ORF/length of full ancestral sequence) of 70%, with very short ancestral sequences treated separately to avoid biases (see STAR Methods). The timing of origination of the ORF was defined as the most ancient ancestor with an intact ORF (some additional criteria were applied, see STAR Methods). In the cases where the most ancient ancestor that could be inferred was intact, the mode of origination of the ORF was "undetermined" (limitation due to absence of more distantly related orthologous regions, or simply because we had reached the root of the tree). However, in the cases where an ancestor lacking an intact ORF was found to precede all ancestors with intact ORFs, the mode of origination of the ORF was deemed as "*de novo*" (Figure 1A).

In total, *de novo* origin was inferred for 155 ORFs. To assess the influence of the length ratio parameter, we tested alternative values: one where *de novo* attribution was stricter (50%) and one more relaxed (80%). The same node of origin was inferred for 102/155 and 148/155 *de novo* ORFs, using the stricter and the more relaxed cutoff respectively (the differences in inferred ages of origin for *de novo* ORFs can be found in Figure S1). Thus, approximately 2/3 of our *de novo* inferred ORFs are entirely robust to this parameter, and for an additional 14 of them, the alternative parameter only changed the date by one or two nodes of the tree.

The presence or absence of transcription in the orthologous region of 92/99 vertebrate species was inferred by examining overlap with annotated transcripts, similarity to known transcript sequences, and by analysis of raw RNA-seq data (see STAR Methods). Transcription was inferred to have originated at the most recent common ancestor of the union of all species in which transcription of the region was detected by any of the three approaches. Comparison of our transcriptional ages to those obtained in two previous studies[44,45] showed that, for the most part, our estimates are at least as ancient as theirs (see Figure S2). Timing of origination of transcript, ORF, as well as all other data gathered for the 715 ORFs can be found in Table S2.

In Figure 1B, we have plotted the distribution of ORF and transcript origination nodes on the vertebrate phylogeny. The origin of the ORF in most cases in the "undetermined mode of origination" category is biased toward the oldest nodes. This result is expected and most likely reflects the limits of homology detection over time due to sequence divergence.[20] The fact that an ORF-first origin (datapoints below the diagonal) is more prevalent for these is probably also an artifact due to our limited capacity to identify distant homologous transcripts. On the other hand, for those cases for which a *de novo* origin can be inferred, we see a prevalence of RNA-first origin for those in the "up – downstream" group. This is to be expected as these ORFs have mostly formed on preexisting mRNAs. For those in the "new – new_iso" group, the situation appears more balanced, with a mix of RNA-first and ORF-first cases. We observe a small number of exceptional cases where an ORF has been sufficiently conserved to allow homology detection since as far back as the split of the Euteleostomi, but we only see evidence for transcription in the human branch. Given that transcript discovery and annotation are far from complete outside of model organisms, it is likely that some of these cases could indeed be false positives, explained by a lack of identified transcripts in species other than human. Alternatively, some could correspond to ORFs that simply happen to overlap with hyper-conserved elements such as enhancers, but that have only recently become transcriptionally active.

We combined the data on the timing of origination of the ORF and the timing of origination of the transcript to infer the timing of origination of the 155 *de novo* origin microproteins. In order for the microprotein to be produced, an ORF and transcription are both necessary, so we define the timing of origination of the microprotein as the earliest node where both are detected (shaded boxes in Figure 1A, henceforth the term "putative origin" will be used for this). An exception is made for cases where transcription evidence postdates the inferred ORF formation but the sequence shows protein-coding conservation signals; in such cases (n = 33), timing of origination of the microprotein is then defined as that of the ORF (see STAR Methods). Note that timing of origination is inferred independently from mode of origination, which can be either "*de novo*" or "undetermined." Lastly but most importantly, while the presence of an ORF and

CellPress
OPEN ACCESS

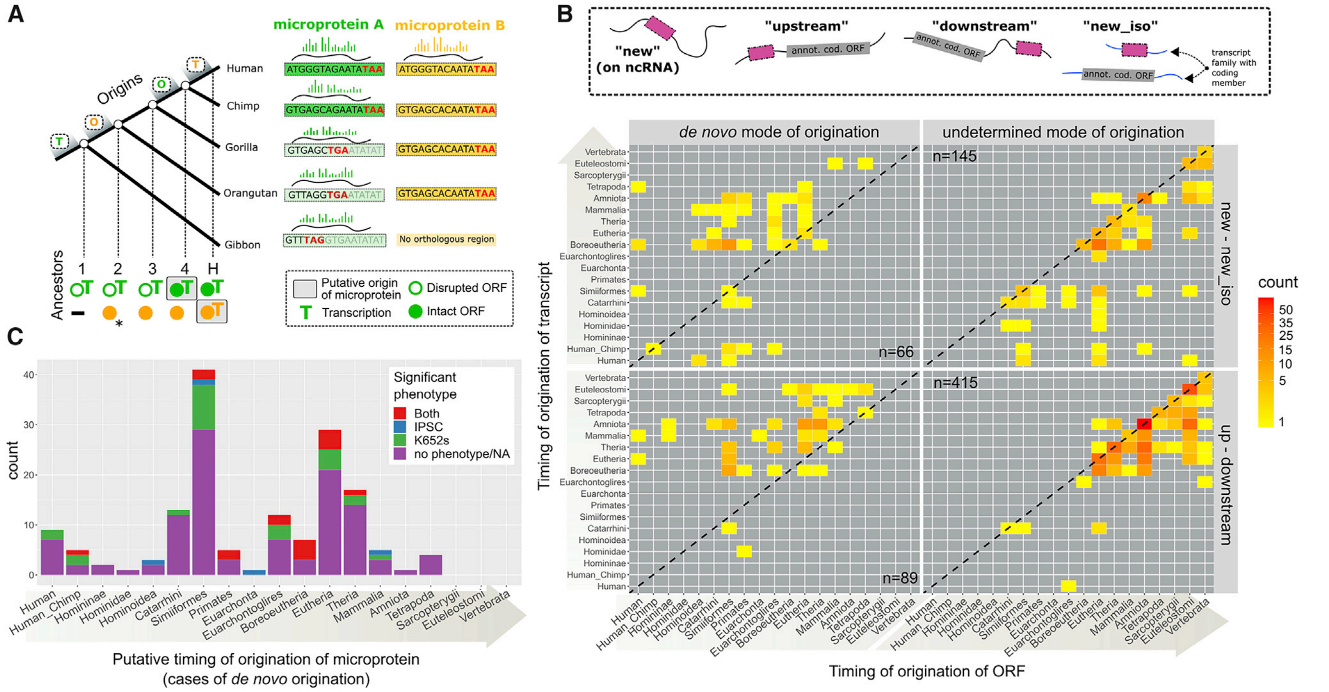

Figure 1. Reconstructing the phylogenetic origins of human microproteins

(A) Graphical example of reconstruction of the timings of origination of the ORF and of transcription for two hypothetical human microproteins. Human ORF A is intact in chimpanzee but disrupted by an early stop codon in the other species. Since the orthologous genomic region has been identified in all four extant species, we can align them and use ancestral sequence reconstruction (ASR) to infer the sequence of all four ancestors and determine whether the ORF is intact or not. In this case, the ORF is not intact (disrupted) in ancestors 1, 2, and 3, since it spans less than 70% of the reconstructed ancestral sequence, and intact in ancestor 4 (reconstructed ancestral sequences are not shown). We can thus infer that the ORF emerged *de novo* and place the node of origin of the ORF (green "O") between ancestors 3 and 4 (for practical purposes, we use the age of ancestor 4). The region orthologous to ORF A has been found to be transcribed in all four species, so the node of origin of transcription is placed at the earliest ancestor (green "T"). The putative origin of the microprotein (gray rectangle) is then calculated as the most recent of the two, which is ancestor 4. ORF B, on the other hand, is intact in all four species where the orthologous region can be identified. ASR estimates that the ORF was intact in ancestors 2, 3, and 4, but no ancestor prior to that one can be inferred. Hence this is a case of "undetermined origin." No transcript has been found in the orthologous region of any of the other species, so transcription is inferred to be human-specific. The putative origin of microprotein B is therefore defined as the human branch, unless there is evidence for protein-coding conservation at the level of the ancestor where the ORF formed, in which case the age of that ancestor would be used as putative origin of microprotein (here, ancestor 2, marked with an asterisk).

(B) Distribution of the phylogenetic origins of ORFs and transcripts in the two broad categories of ORFs. Species and age corresponding to each node of the tree can be found in Figure S3. Nodes are ordered from recent to ancient.

(C) Numbers of *de novo*-originated microproteins with and without significant phenotype in the two cell lines as estimated by Chen et al., grouped by their inferred putative origin. Twenty *de novo* ORFs that have no associated phenotype data are included in the "no phenotype" class.

its transcription are necessary for the expression of a microprotein, they are of course not sufficient. Thus, here we are assuming that every transcribed ORF in species other than human is also translated, an assumption that is bound to be violated. We have consciously made this conservative choice, which means that our estimates of age of origination should be viewed as lower bounds.

Evidence for the biological significance of *de novo* emerged microproteins

The functional relevance of young *de novo* originated ORFs is debated. We thus asked whether any of our recently *de novo* emerged, robustly translated microproteins were found to be functional. For 44/155 *de novo* originated microproteins, CRISPR-Cas-based disruption of their ORF was found to have statistically significant fitness effects in two cell lines (iPSC and K562) according to the strict criteria of Chen et al. This proportion is statistically indistinguishable from that for microproteins of undetermined origin (156/560, X^2 p value = 0.98). The putative origin and knockout phenotype for each of the 155 *de novo* emerged microproteins can be found in Figure 1C.

Our results suggest that there has been ongoing *de novo* birth of functional microproteins since the early evolution of mammals. At least one such microprotein has originated at each of the 13 nodes going back to the mammalian ancestor. The absence in older nodes can be explained by the overall low number of *de novo* genes identified there, which in turn is due to the long evolutionary times. There are 19 *de novo* emerged, functional microproteins that have a putative origin within the past 43 my (since the ancestor of higher primates, Simiiformes). 12 of those are encoded on lncRNAs and seven on coding transcripts. Notably, two of these functional microproteins (CATP00000751060.1 and CATP00001296115.1) have a putative origin after the split of human and chimpanzee. Both are

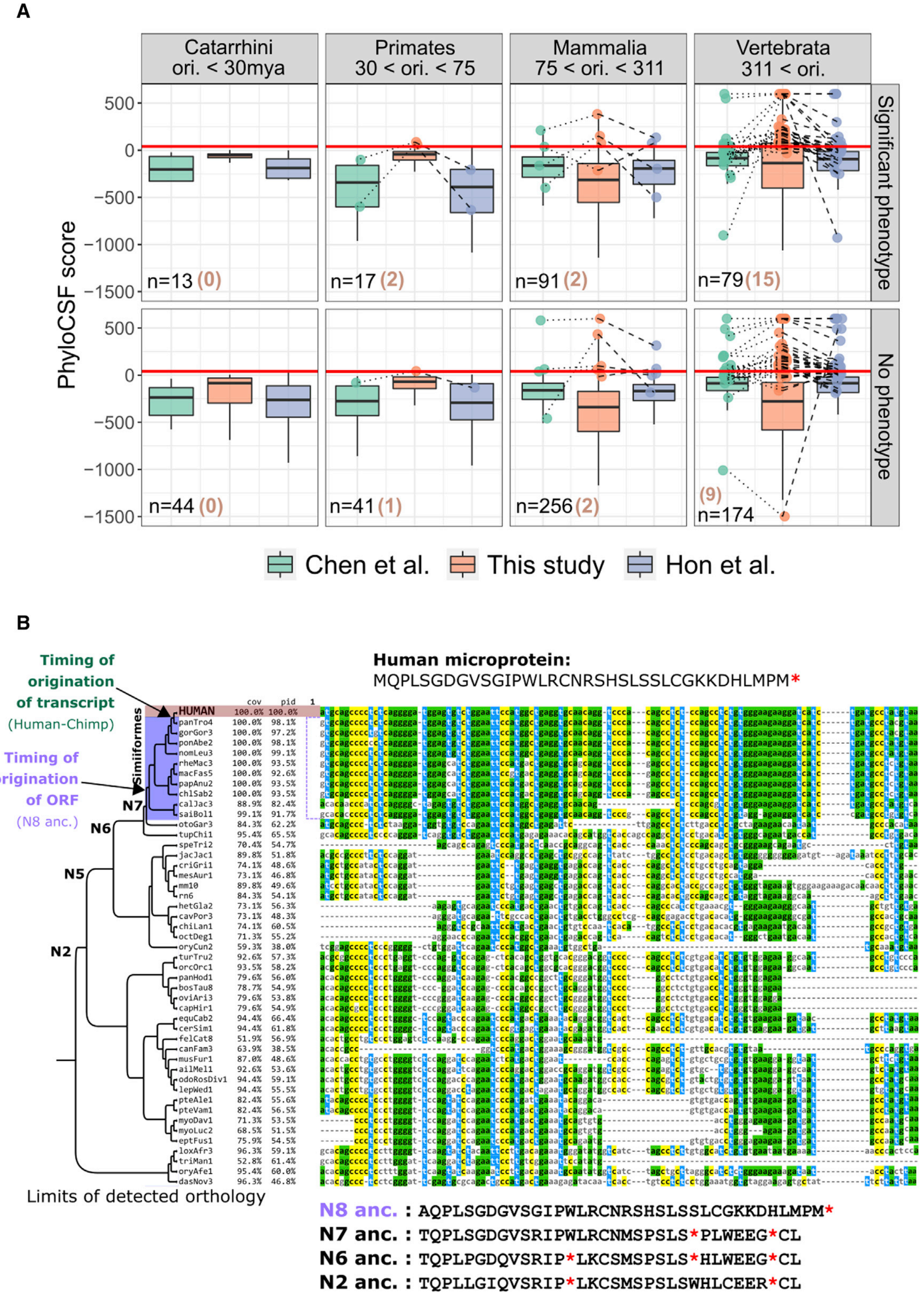

(legend on next page)

expressed from lncRNAs and are cases of ORF-first origination, but with relatively short intervals between the timing of origination of the ORF and that of the human-specific transcript (ORF timings of origination at Hominoidea and Simiiformes). Overall, the results of this analysis provide strong support for the hypothesis that *de novo* emerged microproteins have a ready route to biological significance and may indeed become functional within a relatively short evolutionary time frame.

Numbers of *de novo* originated microproteins with significant phenotypes across ages correlate strongly with those without a phenotype (Spearman's Rho = 0.66, p value = 0.005, excluding the nodes Sarcopterygii, Euteleostomi, and Vertebrata, for which no *de novo* ORFs were found). This implies that a more powerful search for novel microproteins may uncover further functional examples, and that the observed numbers might reflect the limited sampling of cell types and growth conditions experimentally tested.

The fact that some of the microproteins with measurable phenotypes have recently emerged *de novo* and are entirely novel further reinforces the fact that evolutionary conservation and coding signals alone do not reveal the full repertoire of protein-coding genes in a genome. Indeed, out of the 44 *de novo* emerged microproteins with functional evidence, none were predicted as coding by PhyloCSF.[46] This tool, widely used in comparative genomics, determines the likelihood that a sequence is protein-coding based on a nucleotide multiple sequence alignment. It does so by testing whether the alignment best fits a phylogenetic model representing the evolution of codons in known protein-coding genes or one representing the evolution of nucleotide triplet sites in noncoding regions (see Chen et al. and Hon et al.[42,43] and Table S2). None of the 44 *de novo* emerged microproteins were predicted as coding by RNAcode[47] either (Hon et al.[43] and Table S2), and only 4/44 were predicted to be coding based on the ribosome profiling measure (FLOSS score).[43] Only two have a CPAT[48] coding probability higher than 0.5 when calculated over the ORF sequence only (mean of 0.093), and only four are predicted by CPAT to be coding based on analysis of the entire transcript (calculated by Hon et al., see Table S2).

So, are the fitness effects observed really due to the absence of the expressed protein, or could they be coming from the regulatory or RNA level? Chen et al. performed rescue experiments for nine "upstream" and seven "new" ORFs where the ORF peptide was ectopically expressed, as well as controls in which the start codon of the expressed ORF was removed. In all cases, the growth phenotype was rescued, and it was shown that the rescue was dependent on the presence of the start codon. Out of the seven validated "new" ORFs, five are included in our analysis. Only two, with putative origin at Euteleostomi, show signs of being coding (CPAT and PhyloCSF), while the other three are all much more recent with putative origin at Hominoidea (*de novo* mode of origination), Eutheria (undetermined mode of origination), and human (undetermined mode of origination). Similar results are found for "upstream" ORFs. Out of the seven we analyzed, five show coding signatures, and they are all at least as ancient as mammals. The only young one, CATP0000 0415540.1, with a *de novo* origin at the Simiiformes, entirely lacks coding signatures. While more validation experiments will be necessary, these results seem to confirm that the fitness effects of these young, not characteristically coding ORFs are indeed linked to the action of the protein.

Comparative methods such as PhyloCSF should be applied with caution. One difficulty in employing and interpreting PhyloCSF scores in cases such as ours is that failure to recognize the recent *de novo* origin of genes may result in the inclusion of sequences from lineages that diverged before the gene origin and where the ORF is not present. This can negatively bias the coding assessment when the algorithm (correctly) infers a lack of coding potential in a large number of the provided sequences. Frameshifts, which should be more common in evolutionarily recent, less constrained coding sequences, can further complicate coding prediction.

Under this rationale, we hypothesized that considering the phylogenetic origin of each ORF and the conservation of the reading frame in each alignment might ameliorate coding signature detection. We thus applied PhyloCSF in codon-aware alignments comprising only species descending from the predicted node of origin of the ORF (origin of transcription is not taken into account for this specific calculation, see STAR Methods). We then counted the number of ORFs predicted to be coding by each study, taking a frequently used cutoff of PhyloCSF score $\geq$ 41.[43,49] We obtained 62 coding ORFs, that is, 2.8 times more than Chen et al. (22) and 1.8 times more than Hon et al. (35). Half (31/62) are not predicted as coding by either previous study. Importantly, the 31 unique to this study are biased with respect to significant phenotype (19/31, X^2 test, p value = 0.00015). These results, grouped in four broad classes of age of putative origin, are shown in Figure 2A. A similar difference in coding predictions is observed when relaxing the score cutoff to $\geq$10 (75 vs. 42 and 48). Finally, our approach is the only one that identifies any *de novo* originated microprotein with phenotypic effects as coding (3 vs. 0 and 0), arguably the toughest and most critical cases.

Further evidence for the biological significance of a gene can come from an observed association with disease. The disruption of functionally relevant peptides could potentially

Figure 2. Analysis of protein-coding signatures in alignments of human small ORFs and their orthologous loci; example alignment of human ORF CATP00001771233.1

(A) Boxplots show distributions of PhyloCSF scores as calculated in this study, by Hon et al. and by Chen et al. for all 715 microproteins (both *de novo* and undetermined origin) with and without significant phenotypes, (see STAR Methods). Boxplot outliers are not shown. Maximum and minimum values have been set to 600 and –1,500 respectively to improve visualization. Points show ORFs of microproteins that are predicted as coding (score $\geq$ 41, red horizontal line) by at least one study. Lines connect points corresponding to the same ORF. Microproteins are grouped in four broad classes of putative origin age. Numbers in parentheses are coding ORFs uniquely identified as such by our approach.

(B) Phylogenetic tree and multiple sequence alignment of ORF CATP00001771233.1 and its orthologous region in all species where it could be identified. Sequence names correspond to species assembly versions. The translated sequences of human ancestors in the +1 reading frame are shown. N5 ancestor is predicted to be identical to N6. Alignment visualized with Mview,[50] and phylogenetic tree visualized with FigTree. An extended version of this alignment can be found in Data S1.

have pathogenic consequences and even be of clinical importance. To identify such cases, we surveyed all known SNPs annotated as pathogenic or likely pathogenic in dbSNP[51] found within the boundaries of our ORFs' exons.

We identified three such SNPs (summarized in Table S3). ORF CATP00000063293.1 (upstream, *de novo* emerged with a putative origin at Simiiformes) contains one pathogenic SNP (dbSNP: rs1555735545, single nucleotide variant), associated with Limb-girdle muscular dystrophy. The SNP is annotated as an intron/5′ UTR variant, but it does in fact also change the start codon of the encoded protein sequence (ATG → ATA). Consistent with a possible functional role, this ORF has strong PhyloCSF signal, but only when calculated using our ORF-origin-aware approach (88.6 vs. −99, Chen and −205, Hon) and a very high phenotypic score (69, 87th percentile of all ORFs screened by Chen et al.) in K562 cells.

The second pathogenic SNP is found on "new" ORF CATP00000005301.1 (SNV, G>A in the forward strand, dbSNP: rs1238109100). It is tagged as "Likely Pathogenic" related to retinitis pigmentosa.[52] This protein is longer (178 aa), predicted to be entirely disordered, and it too has very high phenotypic score in K562 cells (47.2). It originated in Amniota, and the lncRNA gene is most associated with melanocytes (source: Hon et al.). The mutation would change the 155th amino acid from histidine to tyrosine. Once again, our ORF-origin-aware way of calculating PhyloCSF produces a strong coding prediction (1,959.299), whereas previous estimates had negative scores (−901 Chen, −927 Hon). The strength of this score was surprising, since the microprotein has a more ancient origin than the one described in the previous paragraph. We thus ran PhyloCSF again, on a normal, codon unaware alignment. As expected, the score was strongly negative (−3,062), stressing the importance of reading frame consideration in this type of alignment. CPAT applied on the ORF sequence only also predicts this ORF as coding, with a hexamer log likelihood score of 0.19 (positive values indicate a coding sequence, negative values indicate a noncoding sequence) and a coding probability of 0.85.

The third SNP overlaps ORF CATP00000363722.1 (dbSNP: rs1560929898) and is a single nucleotide deletion that would cause a frameshift after the 16th amino acid. The mutation is associated with Alazami syndrome,[53] which is in line with the ontology association of this lncRNA (embryonic stem cell related according to Hon et al.). Curiously, no significant phenotype or coding signatures were detected for this ORF. Yet we predict an ancient origin (Euteleostomi) and subcellular localization to the mitochondria. Note that, contrary to the first case, the effects of the second and third SNPs could also be due to change in proteins produced by overlapping genes CDH3 and LARP7 (all potential consequences can be found in Table S3). Overall, these three cases provide excellent candidates for further exploration of the clinical significance of novel microproteins.

A novel ORF exemplifies how functional potential can become rapidly fulfilled via *de novo* gene birth

We sought a clear-cut case to exemplify the capacity of *de novo* gene birth to produce a functional protein product in a short evolutionary time frame. CATP00001771233.1 is a 108-nt ORF, found on the intergenic lncRNA RP3-527G5.1 (ENSG00000231811.2; Chen et al. peptide RP3-527G5.1_4347298_36aa), which according to Hon et al. is transcribed through the action of an enhancer (e-lncRNA). The lncRNA gene does not overlap other genes in any strand, with the exception of an intronic region of lncRNA gene ENSG00000285424 (there are however no overlapping exons, see Figure S4A). Multiple sources point to this gene being human specific: Hon et al. detected no orthologous transcription in any tissue in mouse, dog, rat, or chicken, RNAcentral taxonomy results show the transcript as only present in human, and ENSEMBL lists the gene as having zero orthologs and describes it as novel. Nonetheless, our extensive reanalysis of expression data found that the orthologous locus is transcribed in chimpanzee, thus placing the conservative timing of origination of the transcript, and hence of the microprotein, at the human-chimpanzee ancestor.

Based on its reported expression pattern (Hon et al.), the gene is strongly associated with heart tissue (ontology with strongest association is cardiac chamber, followed by cardiac valve, cardiac atrium, melanocyte, atrioventricular valve, and pigment cell, Figure S4B). A very similar expression pattern is found in GTEx for this gene (most expressed in heart, by a large margin, Figure S4C). Consistent with this, in chimpanzee, we only found the locus transcriptionally active in heart tissue and not in any other. No expression in heart was found in gorilla, orangutan, or macaque, where data were available. In human, the gene is also strongly differentially expressed during melanocytic induction, as well as two other experimental series (data not available in other primates).

The identification of the orthologous genomic region that lacks the ORF in species as evolutionarily distant as the armadillo, the results of the ASR, combined with the fact that the protein has no significant matches in any vertebrate proteome (or anywhere else in NCBI's nr database) strongly suggest that this ORF emerged *de novo* (Figure 2B). Our conservative prediction is that the ORF formed at the ancestor of Simiiformes (using a 0.5 length ratio cutoff places the origin slightly earlier, at the ancestor of primates, N6 in Figure 2B). The ATG start codon formed in the human branch, while all other primate species have a GTG codon at that position, which theoretically could still act as a potential start codon (Figure 2B).

No tool predicts coding potential for this ORF or transcript (PhyloCSF Chen et al. score: −327.4246, PhyloCSF Hon et al. score: −318.1374, PhyloCSF our score: −54.3, max CPAT score of transcript: 0.072, CPAT hexamer score for ORF: 0.1, RNAcode p value: 1, sORFs.org FLOSS score: −1), and there is no observable difference in conservation inside and outside of the ORF's exons (Figure S4D). Furthermore, no selection signatures were found, using two additional tools at two phylogenetic levels (all 47 species or 11 primate species) in the reading frame of the microprotein (Table S4, STAR Methods). Interestingly, there are traces of selection coming from the −2 reading frame, in which a longer, overlapping ORF was found (85 aa). We also run phyloP[54] and PhastCons[55] in default mode, two tools that detect conservation in general, regardless of whether the sequence is coding or noncoding. The first found no conservation at either phylogenetic level (both conservation p value > 0.1), while the second found no conservation over all the species in the alignment and only a subset of sites likely under conservation at the primate level (33/108 sites with posterior probability > 0.5).

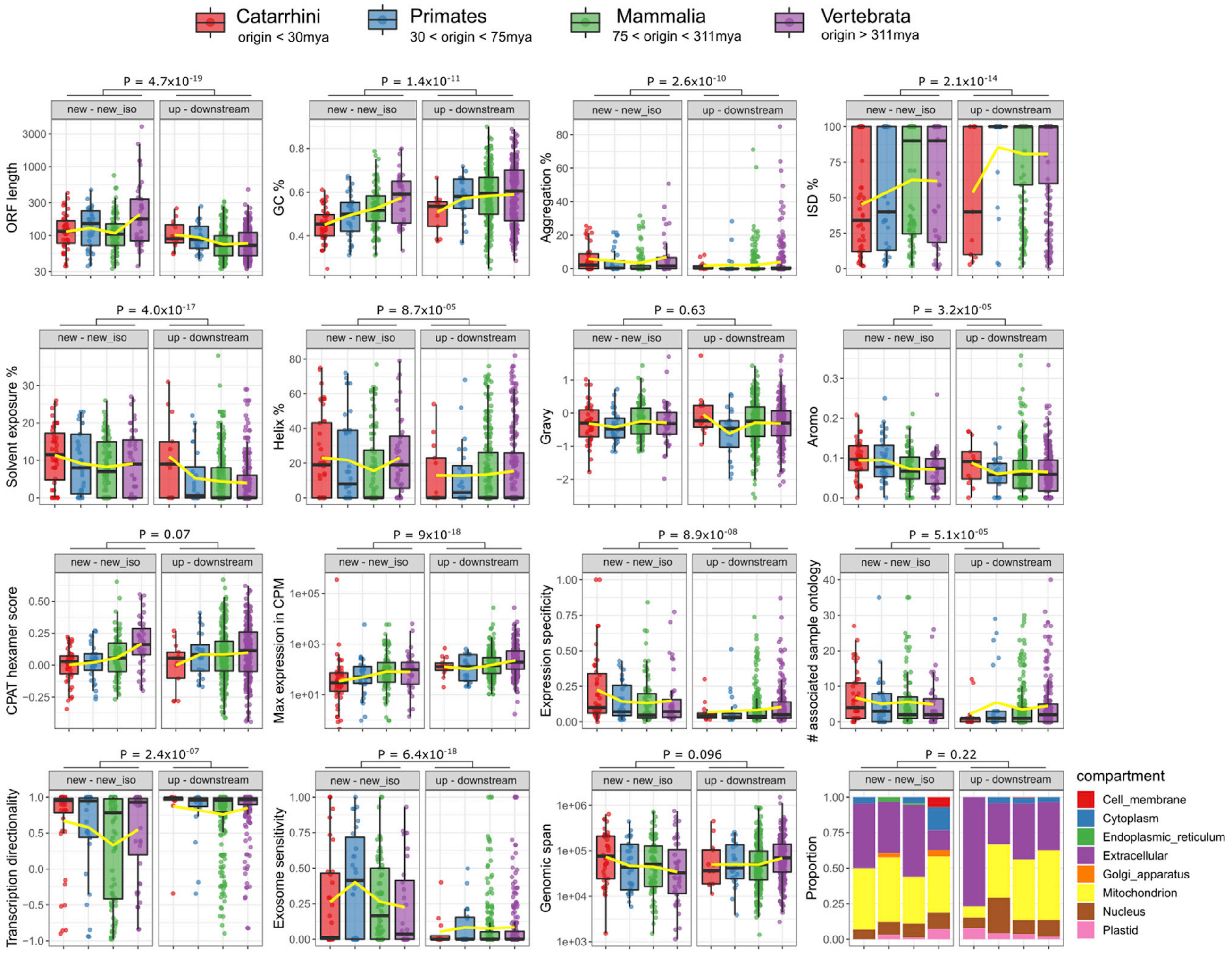

Figure 3. Distributions of various ORF, transcript, and protein properties of human microproteins

Distributions of various ORF, transcript, and protein properties for all 715 microproteins, in four broad groups of putative origin age. Wilcoxon test p values are shown for comparisons of all "new – new_iso" ORFs (n = 211) to all "up – downstream" ones (n = 504), except for subcellular localization (bottom right, X^2 test). Yellow line connects the averages across the groups.

Nonetheless, the ORF is translated with high confidence (ORF-RATER score of 0.85 in iPSC) and has a strong fitness effect in K562 cells (phenotype score: 61.2, 85th percentile). Thus, we are confident both that this is a genuine gene, and that it emerged *de novo*, at the very latest at the human-chimpanzee ancestor.

Given the association with heart, we also sought to confirm expression of this microprotein within a recent dataset of the heart translatome.[56] In this dataset, the transcript is absent in rat and mouse, the only species outside human included in the study. In human, both transcript and ORF show heart-specific expression (RNA only expressed and ORF only translated in iPS-derived cardiomyocytes) further supporting its heart-specific activity. Furthermore, our analysis suggests that the ORF encodes an entirely disordered peptide that has predicted extracellular or nuclear localization. Overall, this example demonstrates that a recently emerged, human ORF can rapidly become functional under a highly specific expression program.

Properties of young and ancient microproteins

In many organisms, it has been shown that evolutionarily novel genes have distinct sequence properties such as low expression and short length.[41] Although our dataset is biased since it only includes unannotated and thus shorter ORFs, we investigated potential differences in various ORF, transcript, and protein properties across four different phylogenetic groups of origin of microproteins (Figure 3).

The most significant difference was observed for GC% between ORFs in the Catarrhini and Vertebrata groups, especially for "new – new_iso" ORFs (avg. GC% 0.45 vs. 0.57, Wilcoxon's test p value = 7.9×10^{-7}). Indeed, there is a correlation between GC% and time since putative origin (Spearman's Rho 0.36, p value = 5×10^{-8}, Figure S5A). The difference is smaller and

statistically weaker for the "up – downstream" class (avg. GC% 0.51 vs. 0.59, Wilcoxon's test p value = 0.036), and there is no correlation of GC% to time since origination (p value = 0.9). Both the difference in the "new – new_iso" class and the absence thereof in the "up – downstream" class are also true when examining entire transcripts (see Figure S5B). A similar trend (young ORFs having lower GC% than older ORFs) was observed by Dowling et al.,[38] albeit between slightly different phylogenetic groups. All these results hold when only using the timing of origination of the ORF as the putative timing of origin of the microprotein, without consideration as to transcription evidence (Figures S6A and S6B).

An equally significant difference was found when comparing the hexamer score (nucleotide hexamer usage bias between coding and noncoding sequences) calculated by CPAT for ORFs. Again, the difference is only found for "new – new_iso" ORFs (average hexamer score 0.0032 vs. 0.17, Wilcoxon's test p value = 1×10^{-5}), and it is absent in the "up – downstream" class (p value = 0.11). This could reflect a tendency in "new" ORFs to become more "gene-like" and more optimized with time. Such a tendency could be absent in "up – downstream" ORFs since they are expected to be enriched in sequence-independent function. Again, these results hold when only taking into account the timing of origination of the ORF (Figure S6A).

Comparing all "new – new_iso" ORFs to all "up – downstream" ones reveals differences in most features we explored. Somewhat expectedly, "new – new_iso" ORFs are longer and GC poorer (albeit that these two properties probably correlate given the fact that start and stop codons are AT-rich[41]; the difference in GC% disappears at the level of entire transcripts, see Figure S5B), the transcripts they are found on are less expressed and with higher tissue specificity, and they have more associated sample ontologies, have lower transcriptional directionality, and are more exosome sensitive. They encode microproteins with higher aggregation propensity, less intrinsic disorder, higher solvent accessibility, higher helix content, and more aromatic residues (Figure 3). Interestingly, only 15 out of the 715 ORFs were found to encode a TM domain showing no timing of origin or significant phenotype bias (X^2 test, both p-values > 0.14). This contrasts with recent findings from budding yeast where the propensity to form transmembrane domains is prevalent among young *de novo* genes.[26] No significant difference was observed in predicted subcellular localization between the various classes.

DISCUSSION

Ribosome profiling has enabled the accurate identification of translated small open reading frames (sORFs). Coupled with experimental evidence for the phenotypic effects of the encoded microproteins, this presents an excellent opportunity to study the evolutionary origin of these elements without being limited to the use of conservation as a proxy for function. Here we explored a set of seemingly functional microproteins and uncovered strong evidence of cases of recent *de novo* origination. For the most part, these lack coding and selection signatures, confirming their novel status.

Our conservative estimate is that 12 such biologically significant microproteins, encoded in lncRNAs (plus another seven on coding transcripts), have arisen *de novo* since the ancestor of all primates, with two being strictly human specific This estimate should be viewed as a strict lower bound since we infer the timing of origination of each microprotein based on the presence of an ORF and the presence of transcription in other species, but not taking into account fitness effects or even translation. This latter one is by definition true for the human ORFs. So, while we treat evidence for presence in the different species equally, it is necessarily true than in many of these cases there will not be a protein translated in other species, and if it is, it might not be functional. Additional, more targeted experimental work is now needed to conclusively demonstrate both the functional role of these elements in human and the absence thereof in other species. Such a confirmation, if it arrives, would be especially consequential for how we annotate functional coding regions in the future. We have no theoretical reason to doubt that a functional microprotein can exist in almost total absence of detectable evolutionary constraints due to its recent origin. This scenario creates a need for models that take such conditions into account. Given how rich the human translatome, transcriptome, and ORFeome are, meeting this need could prove challenging.

An important question is why, out of all the translated novel ORFs, some rapidly acquire biological function while others do not. This is essential to identify the biologically relevant sORFs out of the potentially thousands that could be translated.[5] Will future advances reveal a protogene-model-like reality,[25] where, out of a wide pool of candidates, a few functional ones evolve stochastically? Or could natural selection have acted already to enrich translation of ORFs already more or less primed for functionality, for example by eliminating those likely to be toxic?[57,58] Are these novel microproteins always recruited in a functionally specific manner, or do they initially have more generalized roles? Plausibly, tissue-specific expression of novel peptides might initially carry a smaller risk of overall deleterious effects, thereby potentially "shielding" them from the action of purifying selection. It will also be interesting to understand if, and why, some microproteins evolve in terms of their length, sequence properties, and functional role, while others remain unchanged, resembling frozen accidents.[59]

A related, widely discussed question in the field of *de novo* gene origination is whether the genomic loci out of which novel ORFs emerge have particular characteristics. A high GC% would favor the formation of ORFs, as stop codons are GC poor, but we did not find that young ORFs are more GC rich than ancient ones, rather the opposite. This could be partially explained by a selective pressure for increased GC% (and thus increased expression and/or nuclear export) acting on intronless genes,[60] which are overrepresented among the ORFs studied here (459/715 are single exon). Another possibility is that novel ORFs may emerge out of pseudogenic loci, where longer ORFs once existed but are now defunct. Curiously, the region of CATP00001771233.1, the exemplar ORF above, could correspond to something similar. While there are clearly no traces of selection/conservation on the +1 frame of the sequence (the one encoding the microprotein), the +3 frame produces a positive PhyloCSF score of 48 when focusing on the primates (all scores are strongly negative

CellPress
OPEN ACCESS

using the full alignment). Our dN/dS (ratio of non-synonymous to synonymous substitutions) analysis detected this signal too, but more strongly coming from its reverse complement, −2 frame (see Table S4). We did a search for ORFs on the full sequence of the transcript downloaded from ENSEMBL and found that there are multiple longer overlapping ORFs, including an 85-aa one in the relevant reverse strand. None of these ORFs are found as translated by Chen et al., and three additional ones (apart from CATP00001771233.1) are found translated by van Heesch et al., but these do not correspond to the longer existing ones. More detailed investigation of the genomic region in future could show whether this is a signal from an ancient gene that has become pseudogenized or another novel, overlapping ORF whose translation is yet to be established.

Limitations of the study

The reader should be aware of the various limitations of our work. Perhaps the most significant limitation comes from the inference of phylogenetic ages of transcriptional origins using the presence and absence patterns of transcription in extant species. This entails a non-negligible degree of uncertainty. Because of the patchiness of transcriptional data and the unavoidable limitation in number of tissues and developmental stages sampled, absence of evidence of transcription cannot be equated with evidence of absence; a locus could always be transcribed in a tissue that has yet to be sampled. We have done our best to err on the conservative side and utilize multiple sources of data, and indeed we show that for the most part our estimates are at least as ancient as those of previous studies. Nevertheless, the dynamic nature of transcription, the fast turnover rates of transcripts and their rapid rate of sequence divergence, in addition to the patchiness of the data make it inevitable that we have underestimated the timing of transcriptional origin in some cases. Similarly, inference of the exact phylogenetic branch where an ORF initially formed also comes with uncertainty, as our varying of the cutoff for defining an ORF as intact showed. Future work could reveal that other cutoff values might be more suitable, or that a cutoff tailored to each specific case might be needed, or that a fixed size cutoff applies in general. Conversely, one might speculate that a strict cutoff could turn out to be altogether inappropriate, as there might be no exact evolutionary time point at which an ORF "forms," but rather a gradual transition. More data, coupled with sophisticated phylogenetic models could help elucidate this in the future.

Additionally, the true proportion of young *de novo* ORFs with phenotypic consequences could be higher. Our study is limited in using data from only one series of knockout experiments, but a wider, more comprehensive analysis could bring a more accurate view into focus. This would also increase confidence in the phenotypic effects that are detected, decrease the number of false positives, and allow to more safely build on top of these findings. Given the relatively small size of the dataset, our conclusions about the distribution and properties of functional, *de novo* originated microproteins should be viewed with caution. An additional limitation could stem from the fact that when analyzing the alignments of ORFs to their orthologous regions, we only consider the reading frame corresponding to the human microprotein, and thus we might miss cases of frameshift/overprinting. It is plausible that ASR might also be a source of false positives, in which case some of the ORFs we identified as recently *de novo* emerged could correspond to ancient ones, which through a combination of factors such as sequence divergence, deletions, or chromosomal rearrangements appear recent. Importantly, the use of ASR for the purpose of inferring the origin of an ORF is still in its infancy, and there might be biases at play that could influence our results. For instance, it is known that both alignment quality and branch length uncertainty can directly impact the results of ASR. Application of multiple ASR methodologies under different parameters and thorough assessment of reconstruction uncertainty could in the future alleviate such problems and increase accuracy.

Despite its limitations, our work significantly contributes to our understanding of how protein-coding novelty evolved along the human lineage. It supports a more expansive view of the functional potential of the human genome: a view that embraces these hard-to-detect, small, but significant proteins that show no traces of conservation. Future investigations could determine how many there might exist and reveal their precise role in human physiology and disease.

STAR★METHODS

Detailed methods are provided in the online version of this paper and include the following:

- KEY RESOURCES TABLE
- RESOURCE AVAILABILITY
  - Lead contact
  - Materials availability
  - Data and code availability
- METHOD DETAILS
  - Data collection
  - RNA-seq data processing and mapping
  - Improvement of transcript set and expression quantification with StringTie
  - Inference of orthologous transcription based on reference transcriptomes
  - Inference of orthologous transcription based on analysis of expression data
  - Validation of timing or origination of transcript using other transcriptome sources
  - Identification of orthologous genomic regions and inference of presence of ancestral ORFs
  - Functional signatures and statistics
- QUANTIFICATION AND STATISTICAL ANALYSIS

SUPPLEMENTAL INFORMATION

Supplemental information can be found online at https://doi.org/10.1016/j.celrep.2022.111808.

ACKNOWLEDGMENTS

We are grateful to Jin Chen for providing us with data and methodological details.[42] We also thank Laurence Hurst, Anthony Redmond, and members of the Carvunis lab for valuable feedback on the manuscript. This work was supported by funding from the European Research Council, grant agreement

771419. This research is co-financed by Greece and the European Union (European Social Fund, ESF) through the Operational Program "Human Resources Development, Education and Lifelong Learning" in the context of the project "Reinforcement of Postdoctoral Researchers – second Cycle" (MIS-5033021), implemented by the State Scholarships Foundation (IKU).

AUTHOR CONTRIBUTIONS

A.McL. and N.V. conceived the study. N.V. and Z.V. performed the analyses. N.V., Z.V., and K.D. analyzed the data. A.McL., N.V. and Z.V. wrote the paper. All authors read, finalized, and approved the final manuscript.

DECLARATION OF INTERESTS

A.McL. was a member of the journal's Advisory Board at the time of this article's initial submission.

Received: October 18, 2021
Revised: June 21, 2022
Accepted: November 18, 2022
Published: December 20, 2022

REFERENCES

1. Ingolia, N.T., Brar, G.A., Stern-Ginossar, N., Harris, M.S., Talhouarne, G.J.S., Jackson, S.E., Wills, M.R., and Weissman, J.S. (2014). Ribosome profiling reveals pervasive translation outside of annotated protein-coding genes. Cell Rep. *8*, 1365–1379. https://doi.org/10.1016/j.celrep.2014.07.045.
2. Andrews, S.J., and Rothnagel, J.A. (2014). Emerging evidence for functional peptides encoded by short open reading frames. Nat. Rev. Genet. *15*, 193–204. https://doi.org/10.1038/nrg3520.
3. Calvo, S.E., Pagliarini, D.J., and Mootha, V.K. (2009). Upstream open reading frames cause widespread reduction of protein expression and are polymorphic among humans. Proc. Natl. Acad. Sci. USA *106*, 7507–7512. https://doi.org/10.1073/pnas.0810916106.
4. Makarewich, C.A. (2020). The hidden world of membrane microproteins. Exp. Cell Res. *388*, 111853. https://doi.org/10.1016/j.yexcr.2020.111853.
5. Couso, J.-P., and Patraquim, P. (2017). Classification and function of small open reading frames. Nat. Rev. Mol. Cell Biol. *18*, 575–589. https://doi.org/10.1038/nrm.2017.58.
6. Schlesinger, D., and Elsässer, S.J. (2022). Revisiting sORFs: overcoming challenges to identify and characterize functional microproteins. FEBS J. *289*, 53–74. https://doi.org/10.1111/febs.15769.
7. Prensner, J.R., Enache, O.M., Luria, V., Krug, K., Clauser, K.R., Dempster, J.M., Karger, A., Wang, L., Stumbraite, K., Wang, V.M., et al. (2021). Noncanonical open reading frames encode functional proteins essential for cancer cell survival. Nat. Biotechnol. *39*, 697–704. https://doi.org/10.1038/s41587-020-00806-2.
8. Rathore, A., Martinez, T.F., Chu, Q., and Saghatelian, A. (2018). Small, but mighty? Searching for human microproteins and their potential for understanding health and disease. Expert Rev. Proteomics *15*, 963–965. https://doi.org/10.1080/14789450.2018.1547194.
9. D'Lima, N.G., Ma, J., Winkler, L., Chu, Q., Loh, K.H., Corpuz, E.O., Budnik, B.A., Lykke-Andersen, J., Saghatelian, A., and Slavoff, S.A. (2017). A human microprotein that interacts with the mRNA decapping complex. Nat. Chem. Biol. *13*, 174–180. https://doi.org/10.1038/nchembio.2249.
10. Chu, Q., Martinez, T.F., Novak, S.W., Donaldson, C.J., Tan, D., Vaughan, J.M., Chang, T., Diedrich, J.K., Andrade, L., Kim, A., et al. (2019). Regulation of the ER stress response by a mitochondrial microprotein. Nat. Commun. *10*, 4883–4913. https://doi.org/10.1038/s41467-019-12816-z.
11. Anderson, D.M., Anderson, K.M., Chang, C.-L., Makarewich, C.A., Nelson, B.R., McAnally, J.R., Kasaragod, P., Shelton, J.M., Liou, J., Bassel-Duby, R., and Olson, E.N. (2015). A micropeptide encoded by a putative long non-coding RNA regulates muscle performance. Cell *160*, 595–606. https://doi.org/10.1016/j.cell.2015.01.009.
12. Zhang, Q., Vashisht, A.A., O'Rourke, J., Corbel, S.Y., Moran, R., Romero, A., Miraglia, L., Zhang, J., Durrant, E., Schmedt, C., et al. (2017). The microprotein Minion controls cell fusion and muscle formation. Nat. Commun. *8*, 15664. https://doi.org/10.1038/ncomms15664.
13. Graeff, M., Straub, D., Eguen, T., Dolde, U., Rodrigues, V., Brandt, R., and Wenkel, S. (2016). MicroProtein-mediated recruitment of CONSTANS into a TOPLESS trimeric complex represses flowering in arabidopsis. PLoS Genet. *12*, e1005959. https://doi.org/10.1371/journal.pgen.1005959.
14. Miravet-Verde, S., Ferrar, T., Espadas-García, G., Mazzolini, R., Gharrab, A., Sabido, E., Serrano, L., and Lluch-Senar, M. (2019). Unraveling the hidden universe of small proteins in bacterial genomes. Mol. Syst. Biol. *15*, e8290. https://doi.org/10.15252/msb.20188290.
15. Storz, G., Wolf, Y.I., and Ramamurthi, K.S. (2014). Small proteins can No longer Be ignored. Annu. Rev. Biochem. *83*, 753–777. https://doi.org/10.1146/annurev-biochem-070611-102400.
16. Mackowiak, S.D., Zauber, H., Bielow, C., Thiel, D., Kutz, K., Calviello, L., Mastrobuoni, G., Rajewsky, N., Kempa, S., Selbach, M., and Obermayer, B. (2015). Extensive identification and analysis of conserved small ORFs in animals. Genome Biol. *16*, 179. https://doi.org/10.1186/s13059-015-0742-x.
17. Straub, D., and Wenkel, S. (2017). Cross-species genome-wide identification of evolutionary conserved MicroProteins. Genome Biol. Evol. *9*, 777–789. https://doi.org/10.1093/gbe/evx041.
18. Magny, E.G., Pueyo, J.I., Pearl, F.M.G., Cespedes, M.A., Niven, J.E., Bishop, S.A., and Couso, J.P. (2013). Conserved regulation of cardiac calcium uptake by peptides encoded in small open reading frames. Science *341*, 1116–1120. https://doi.org/10.1126/science.1238802.
19. Ruiz-Orera, J., and Albà, M.M. (2019). Translation of small open reading frames: roles in regulation and evolutionary innovation. Trends Genet. *35*, 186–198. https://doi.org/10.1016/j.tig.2018.12.003.
20. Vakirlis, N., Carvunis, A.-R., and McLysaght, A. (2020). Synteny-based analyses indicate that sequence divergence is not the main source of orphan genes. Elife *9*, e53500. https://doi.org/10.7554/eLife.53500.
21. Andersson, D.I., Jerlström-Hultqvist, J., and Näsvall, J. (2015). Evolution of new functions de novo and from preexisting genes. Cold Spring Harbor Perspect. Biol. *7*, a017996. https://doi.org/10.1101/cshperspect.a017996.
22. Tautz, D., and Domazet-Lošo, T. (2011). The evolutionary origin of orphan genes. Nat. Rev. Genet. *12*, 692–702. https://doi.org/10.1038/nrg3053.
23. Van Oss, S.B., and Carvunis, A.-R. (2019). De novo gene birth. PLoS Genet. *15*, e1008160. https://doi.org/10.1371/journal.pgen.1008160.
24. Vakirlis, N., Hebert, A.S., Opulente, D.A., Achaz, G., Hittinger, C.T., Fischer, G., Coon, J.J., and Lafontaine, I. (2018). A molecular portrait of de novo genes in yeasts. Mol. Biol. Evol. *35*, 631–645. https://doi.org/10.1093/molbev/msx315.
25. Carvunis, A.-R., Rolland, T., Wapinski, I., Calderwood, M.A., Yildirim, M.A., Simonis, N., Charloteaux, B., Hidalgo, C.A., Barbette, J., Santhanam, B., et al. (2012). Proto-genes and de novo gene birth. Nature *487*, 370–374. https://doi.org/10.1038/nature11184.
26. Vakirlis, N., Acar, O., Hsu, B., Castilho Coelho, N., Van Oss, S.B., Wacholder, A., Medetgul-Ernar, K., Bowman, R.W., Hines, C.P., Iannotta, J., et al. (2020). De novo emergence of adaptive membrane proteins from thymine-rich genomic sequences. Nat. Commun. *11*, 781–818. https://doi.org/10.1038/s41467-020-14500-z.
27. Xie, C., Bekpen, C., Künzel, S., Keshavarz, M., Krebs-Wheaton, R., Skrabar, N., Ullrich, K.K., and Tautz, D. (2019). A de novo evolved gene in the house mouse regulates female pregnancy cycles. Elife *8*, e44392. https://doi.org/10.7554/eLife.44392.
28. Heames, B., Schmitz, J., and Bornberg-Bauer, E. (2020). A continuum of evolving de novo genes drives protein-coding novelty in Drosophila. J. Mol. Evol. *88*, 382–398. https://doi.org/10.1007/s00239-020-09939-z.
29. Schmitz, J.F., Chain, F.J.J., and Bornberg-Bauer, E. (2020). Evolution of novel genes in three-spined stickleback populations. Heredity *125*, 50–59. https://doi.org/10.1038/s41437-020-0319-7.

CellPress
OPEN ACCESS

30. Zhuang, X., Yang, C., Murphy, K.R., and Cheng, C.-H.C. (2019). Molecular mechanism and history of non-sense to sense evolution of antifreeze glycoprotein gene in northern gadids. Proc. Natl. Acad. Sci. USA *116*, 4400–4405. https://doi.org/10.1073/pnas.1817138116.

31. Zhang, L., Ren, Y., Yang, T., Li, G., Chen, J., Gschwend, A.R., Yu, Y., Hou, G., Zi, J., Zhou, R., et al. (2019). Rapid evolution of protein diversity by de novo origination in Oryza. Nat. Ecol. Evol. *3*, 679–690. https://doi.org/10.1038/s41559-019-0822-5.

32. Prabh, N., Roeseler, W., Witte, H., Eberhardt, G., Sommer, R.J., and Rödelsperger, C. (2018). Deep taxon sampling reveals the evolutionary dynamics of novel gene families in Pristionchus nematodes. Genome Res. *28*, 1664–1674. https://doi.org/10.1101/gr.234971.118.

33. Knowles, D.G., and McLysaght, A. (2009). Recent de novo origin of human protein-coding genes. Genome Res. *19*, 1752–1759. https://doi.org/10.1101/gr.095026.109.

34. Wu, D.-D., Irwin, D.M., and Zhang, Y.-P. (2011). De novo origin of human protein-coding genes. PLoS Genet. *7*, e1002379. https://doi.org/10.1371/journal.pgen.1002379.

35. Chen, J.-Y., Shen, Q.S., Zhou, W.-Z., Peng, J., He, B.Z., Li, Y., Liu, C.-J., Luan, X., Ding, W., Li, S., et al. (2015). Emergence, retention and selection: a trilogy of origination for functional de novo proteins from ancestral LncRNAs in primates. PLoS Genet. *11*, e1005391. https://doi.org/10.1371/journal.pgen.1005391.

36. Toll-Riera, M., Castelo, R., Bellora, N., and Albà, M.M. (2009). Evolution of primate orphan proteins. Biochem. Soc. Trans. *37*, 778–782. https://doi.org/10.1042/BST0370778.

37. Ruiz-Orera, J., Hernandez-Rodriguez, J., Chiva, C., Sabidó, E., Kondova, I., Bontrop, R., Marqués-Bonet, T., and Albà, M.M. (2015). Origins of de novo genes in human and chimpanzee. PLoS Genet. *11*, e1005721. https://doi.org/10.1371/journal.pgen.1005721.

38. Dowling, D., Schmitz, J.F., and Bornberg-Bauer, E. (2020). Stochastic gain and loss of novel transcribed open reading frames in the human lineage. Genome Biol. Evol. *12*, 2183–2195. https://doi.org/10.1093/gbe/evaa194.

39. Cai, J., Zhao, R., Jiang, H., and Wang, W. (2008). De novo origination of a new protein-coding gene in Saccharomyces cerevisiae. Genetics *179*, 487–496. https://doi.org/10.1534/genetics.107.084491.

40. Nielly-Thibault, L., and Landry, C.R. (2019). Differences between the raw material and the products of de Novo gene birth can result from mutational biases. Genetics *212*, 1353–1366. https://doi.org/10.1534/genetics.119.302187.

41. McLysaght, A., and Hurst, L.D. (2016). Open questions in the study of de novo genes: what, how and why. Nat. Rev. Genet. *17*, 567–578. https://doi.org/10.1038/nrg.2016.78.

42. Chen, J., Brunner, A.-D., Cogan, J.Z., Nuñez, J.K., Fields, A.P., Adamson, B., Itzhak, D.N., Li, J.Y., Mann, M., Leonetti, M.D., and Weissman, J.S. (2020). Pervasive functional translation of noncanonical human open reading frames. Science *367*, 1140–1146. https://doi.org/10.1126/science.aay0262.

43. Hon, C.-C., Ramilowski, J.A., Harshbarger, J., Bertin, N., Rackham, O.J.L., Gough, J., Denisenko, E., Schmeier, S., Poulsen, T.M., Severin, J., et al. (2017). An atlas of human long non-coding RNAs with accurate 5′ ends. Nature *543*, 199–204. https://doi.org/10.1038/nature21374.

44. Necsulea, A., Soumillon, M., Warnefors, M., Liechti, A., Daish, T., Zeller, U., Baker, J.C., Grützner, F., and Kaessmann, H. (2014). The evolution of lncRNA repertoires and expression patterns in tetrapods. Nature *505*, 635–640. https://doi.org/10.1038/nature12943.

45. Sarropoulos, I., Marin, R., Cardoso-Moreira, M., and Kaessmann, H. (2019). Developmental dynamics of lncRNAs across mammalian organs and species. Nature *571*, 510–514. https://doi.org/10.1038/s41586-019-1341-x.

46. Lin, M.F., Jungreis, I., and Kellis, M. (2011). PhyloCSF: a comparative genomics method to distinguish protein coding and non-coding regions. Bioinformatics *27*, i275–i282. https://doi.org/10.1093/bioinformatics/btr209.

47. Washietl, S., Findeiss, S., Müller, S.A., Kalkhof, S., von Bergen, M., Hofacker, I.L., Stadler, P.F., and Goldman, N. (2011). RNAcode: robust discrimination of coding and noncoding regions in comparative sequence data. RNA *17*, 578–594. https://doi.org/10.1261/rna.2536111.

48. Wang, L., Park, H.J., Dasari, S., Wang, S., Kocher, J.-P., and Li, W. (2013). CPAT: coding-Potential Assessment Tool using an alignment-free logistic regression model. Nucleic Acids Res. *41*, e74. https://doi.org/10.1093/nar/gkt006.

49. Volders, P.-J., Verheggen, K., Menschaert, G., Vandepoele, K., Martens, L., Vandesompele, J., and Mestdagh, P. (2015). An update on LNCipedia: a database for annotated human lncRNA sequences. Nucleic Acids Res. *43*, D174–D180. https://doi.org/10.1093/nar/gku1060.

50. Madeira, F., Park, Y.M., Lee, J., Buso, N., Gur, T., Madhusoodanan, N., Basutkar, P., Tivey, A.R.N., Potter, S.C., Finn, R.D., and Lopez, R. (2019). The EMBL-EBI search and sequence analysis tools APIs in 2019. Nucleic Acids Res. *47*, W636–W641. https://doi.org/10.1093/nar/gkz268.

51. Sherry, S.T., Ward, M.-H., Kholodov, M., Baker, J., Phan, L., Smigielski, E.M., and Sirotkin, K. (2001). dbSNP: the NCBI database of genetic variation. Nucleic Acids Res. *29*, 308–311. https://doi.org/10.1093/nar/29.1.308.

52. Jespersgaard, C., Fang, M., Bertelsen, M., Dang, X., Jensen, H., Chen, Y., Bech, N., Dai, L., Rosenberg, T., Zhang, J., et al. (2019). Molecular genetic analysis using targeted NGS analysis of 677 individuals with retinal dystrophy. Sci. Rep. *9*, 1219. https://doi.org/10.1038/s41598-018-38007-2.

53. Bertoli-Avella, A.M., Beetz, C., Ameziane, N., Rocha, M.E., Guatibonza, P., Pereira, C., Calvo, M., Herrera-Ordonez, N., Segura-Castel, M., Diego-Alvarez, D., et al. (2021). Successful application of genome sequencing in a diagnostic setting: 1007 index cases from a clinically heterogeneous cohort. Eur. J. Hum. Genet. *29*, 141–153. https://doi.org/10.1038/s41431-020-00713-9.

54. Hubisz, M.J., Pollard, K.S., and Siepel, A. (2011). PHAST and RPHAST: phylogenetic analysis with space/time models. Brief. Bioinform. *12*, 41–51. https://doi.org/10.1093/bib/bbq072.

55. Siepel, A., Bejerano, G., Pedersen, J.S., Hinrichs, A.S., Hou, M., Rosenbloom, K., Clawson, H., Spieth, J., Hillier, L.W., Richards, S., et al. (2005). Evolutionarily conserved elements in vertebrate, insect, worm, and yeast genomes. Genome Res *15*, 1034–1050. https://doi.org/10.1101/gr.3715005.

56. van Heesch, S., Witte, F., Schneider-Lunitz, V., Schulz, J.F., Adami, E., Faber, A.B., Kirchner, M., Maatz, H., Blachut, S., Sandmann, C.-L., et al. (2019). The translational landscape of the human heart. Cell *178*, 242–260.e29. https://doi.org/10.1016/j.cell.2019.05.010.

57. Wilson, B.A., and Masel, J. (2011). Putatively noncoding transcripts show extensive association with ribosomes. Genome Biol. Evol. *3*, 1245–1252. https://doi.org/10.1093/gbe/evr099.

58. Kosinski, L.J., and Masel, J. (2020). Readthrough errors purge deleterious cryptic sequences, facilitating the birth of coding sequences. Mol. Biol. Evol. *37*, 1761–1774. https://doi.org/10.1093/molbev/msaa046.

59. Schmitz, J.F., Ullrich, K.K., and Bornberg-Bauer, E. (2018). Incipient de novo genes can evolve from frozen accidents that escaped rapid transcript turnover. Nat. Ecol. Evol. *2*, 1626–1632. https://doi.org/10.1038/s41559-018-0639-7.

60. Mordstein, C., Savisaar, R., Young, R.S., Bazile, J., Talmane, L., Luft, J., Liss, M., Taylor, M.S., Hurst, L.D., and Kudla, G. (2020). Codon usage and splicing jointly influence mRNA localization. Cell Syst. *10*, 351–362.e8. https://doi.org/10.1016/j.cels.2020.03.001.

61. Wang, Z.-Y., Leushkin, E., Liechti, A., Ovchinnikova, S., Mößinger, K., Brüning, T., Rummel, C., Grützner, F., Cardoso-Moreira, M., Janich, P., et al. (2020). Transcriptome and translatome co-evolution in mammals. Nature *588*, 642–647. https://doi.org/10.1038/s41586-020-2899-z.

62. Brawand, D., Soumillon, M., Necsulea, A., Julien, P., Csárdi, G., Harrigan, P., Weier, M., Liechti, A., Aximu-Petri, A., Kircher, M., et al. (2011). The evolution of gene expression levels in mammalian organs. Nature *478*, 343–348. https://doi.org/10.1038/nature10532.

63. Roller, M., Stamper, E., Villar, D., Izuogu, O., Martin, F., Redmond, A.M., Ramachanderan, R., Harewood, L., Odom, D.T., and Flicek, P. (2021). LINE retrotransposons characterize mammalian tissue-specific and evolutionarily dynamic regulatory regions. Genome Biol. *22*, 62. https://doi.org/10.1186/s13059-021-02260-y.

64. Wang, S., Li, W., Liu, S., and Xu, J. (2016). RaptorX-Property: a web server for protein structure property prediction. Nucleic Acids Res. *44*, W430–W435. https://doi.org/10.1093/nar/gkw306.

65. Käll, L., Krogh, A., and Sonnhammer, E.L.L. (2007). Advantages of combined transmembrane topology and signal peptide prediction—the Phobius web server. Nucleic Acids Res. *35*, W429–W432. https://doi.org/10.1093/nar/gkm256.

66. Dosztányi, Z., Csizmok, V., Tompa, P., and Simon, I. (2005). IUPred: web server for the prediction of intrinsically unstructured regions of proteins based on estimated energy content. Bioinformatics *21*, 3433–3434. https://doi.org/10.1093/bioinformatics/bti541.

67. Almagro Armenteros, J.J., Sønderby, C.K., Sønderby, S.K., Nielsen, H., and Winther, O. (2017). DeepLoc: prediction of protein subcellular localization using deep learning. Bioinformatics *33*, 3387–3395. https://doi.org/10.1093/bioinformatics/btx431.

68. Quinlan, A.R. (2014). BEDTools: the Swiss-army tool for genome feature analysis. Curr. Protoc. Bioinformatics *47*, 11.12.1.11.12.34. https://doi.org/10.1002/0471250953.bi1112s47.

69. Peden, J. Correspondence analysis of codon usage. http://codonw.sourceforge.net/.

70. Martin, M. (2011). Cutadapt removes adapter sequences from high-throughput sequencing reads. EMBnet. j. *17*, 10–12. https://doi.org/10.14806/ej.17.1.200.

71. Dobin, A., Davis, C.A., Schlesinger, F., Drenkow, J., Zaleski, C., Jha, S., Batut, P., Chaisson, M., and Gingeras, T.R. (2013). STAR: ultrafast universal RNA-seq aligner. Bioinformatics *29*, 15–21. https://doi.org/10.1093/bioinformatics/bts635.

72. Pertea, M., Pertea, G.M., Antonescu, C.M., Chang, T.-C., Mendell, J.T., and Salzberg, S.L. (2015). StringTie enables improved reconstruction of a transcriptome from RNA-seq reads. Nat. Biotechnol. *33*, 290–295. https://doi.org/10.1038/nbt.3122.

73. Altschul, S.F., Madden, T.L., Schäffer, A.A., Zhang, J., Zhang, Z., Miller, W., and Lipman, D.J. (1997). Gapped BLAST and PSI-BLAST: a new generation of protein database search programs. Nucleic Acids Res. *25*, 3389–3402.

74. Katoh, K., and Standley, D.M. (2013). MAFFT multiple sequence alignment software version 7: improvements in performance and usability. Mol. Biol. Evol. *30*, 772–780. https://doi.org/10.1093/molbev/mst010.

75. Lemoine, F., and Gascuel, O. (2021). Gotree/Goalign: toolkit and Go API to facilitate the development of phylogenetic workflows. NAR Genom. Bioinform. *3*, lqab075. https://doi.org/10.1093/nargab/lqab075.

76. Kozlov, A.M., Darriba, D., Flouri, T., Morel, B., and Stamatakis, A. (2019). RAxML-NG: a fast, scalable and user-friendly tool for maximum likelihood phylogenetic inference. Bioinformatics *35*, 4453–4455. https://doi.org/10.1093/bioinformatics/btz305.

77. Ashkenazy, H., Penn, O., Doron-Faigenboim, A., Cohen, O., Cannarozzi, G., Zomer, O., and Pupko, T. (2012). FastML: a web server for probabilistic reconstruction of ancestral sequences. Nucleic Acids Res. *40*, W580–W584. https://doi.org/10.1093/nar/gks498.

78. Abascal, F., Zardoya, R., and Telford, M.J. (2010). TranslatorX: multiple alignment of nucleotide sequences guided by amino acid translations. Nucleic Acids Res. *38*, W7–13. https://doi.org/10.1093/nar/gkq291.

79. HyPhy: Hypothesis Testing Using Phylogenies, SpringerLink. https://link.springer.com/chapter/10.1007%2F0-387-27733-1_6.

80. Yang, Z. (2007). Paml 4: phylogenetic analysis by maximum likelihood. Mol. Biol. Evol. *24*, 1586–1591. https://doi.org/10.1093/molbev/msm088.

81. Fields, A.P., Rodriguez, E.H., Jovanovic, M., Stern-Ginossar, N., Haas, B.J., Mertins, P., Raychowdhury, R., Hacohen, N., Carr, S.A., Ingolia, N.T., et al. (2015). A regression-based analysis of ribosome-profiling data reveals a conserved complexity to mammalian translation. Mol. Cell *60*, 816–827. https://doi.org/10.1016/j.molcel.2015.11.013.

82. Hedges, S.B., Dudley, J., and Kumar, S. (2006). TimeTree: a public knowledge-base of divergence times among organisms. Bioinformatics *22*, 2971–2972. https://doi.org/10.1093/bioinformatics/btl505.

83. Wickham, H. (2011). ggplot2. WIREs. Comp. Stat. *3*, 180–185. https://doi.org/10.1002/wics.147.

STAR★METHODS

KEY RESOURCES TABLE

REAGENT or RESOURCE	SOURCE	IDENTIFIER
Deposited data		
Translation and functional data of ORFs	Chen et al.[42]	Supplemental tables
Human transcript and ORF data	FANTOM-CAT (Hon et al.[43])	https://fantom.gsc.riken.jp/5/suppl/Hon_et_al_2016/data/
Vertebrate RefSeq transcript sequence database	NCBI's RefSeq	https://ftp.ncbi.nlm.nih.gov/refseq/release/vertebrate_mammalian/ https://ftp.ncbi.nlm.nih.gov/refseq/release/vertebrate_other/
100-way vertebrate genome alignment	UCSC genome browser	https://hgdownload.soe.ucsc.edu/downloads.html
Raw RNA-seq data from human tissue samples: Brain, Cerebellum, Heart, Kidney, Liver, Ovary, Placenta, Testis	Wang et al.[61]; Brawand et al.[62]; Necsulea et al.[44]	ERR2812349, ERR2812350, ERR2812351, SRR306844, SRR306845, SRR306846, SRR306847, SRR306848, SRR306849, SRR306850, SRR306851, SRR306852, SRR306853, ERR2812355, ERR2812356, ERR2812357, SRR649364, SRR943341, SRR649363, SRR943340, SRR943354, SRR943359, ERR2812361, ERR2812362, ERR2812363
Raw RNA-seq data from chimp tissue samples: Brain, Cerebellum, Heart, Kidney, Liver, Testis	Brawand et al.[62]	SRR306811, SRR306812, SRR306813, SRR306814, SRR306815, SRR306816, SRR306817, SRR306818, SRR306819, SRR306820, SRR306821, SRR306822, SRR306823, SRR306824, SRR306825
Raw RNA-seq data from gorilla tissue samples: Brain, Cerebellum, Heart, Kidney, Liver, Testis	Brawand et al.[62]	SRR306800, SRR306801, SRR306802, SRR306803, SRR306804, SRR306805, SRR306806, SRR306807, SRR306808, SRR306809, SRR306810
Raw RNA-seq data from orangutan tissue samples: Brain, Cerebellum, Heart, Kidney, Liver	Brawand et al.[62]	SRR306791, SRR306792, SRR306793, SRR306794, SRR306795, SRR306796, SRR306797, SRR306798, SRR306799
Raw RNA-seq data from macaque tissue samples: Brain, Cerebellum, Heart, Kidney, Liver, Muscle, Testis	Wang et al.[61]; Brawand et al.[62]; Roller et al.[63]	ERR2812367, ERR2812368, ERR2812369, SRR306780, SRR306781, SRR306782, SRR306783, SRR306784, SRR306785, ERR2812373, ERR2812374, ERR2812375, ERR3417964, ERR3417996, ERR3418000, ERR2812379, ERR2812380, ERR2812381

(*Continued on next page*)

Continued

REAGENT or RESOURCE	SOURCE	IDENTIFIER
Raw RNA-seq data from marmoset tissue samples: Brain, Liver, Muscle, Testis	Roller et al.[63]	ERR3417915, ERR3417984, ERR3417986, ERR3417938, ERR3417970, ERR3417975, ERR3417914, ERR3417968, ERR3417987, ERR3417930, ERR3417944, ERR3417962
Raw RNA-seq data from mouse tissue samples: Brain, Cerebellum, Heart, Kidney, Liver, Muscle, Ovary, Placenta, Testis	Wang et al.[61]; Brawand et al.[62]; Roller et al.[63]; Necsulea et al.[44]	ERR2812385, ERR2812386, ERR2812387, SRR306763, SRR306764, SRR306765, SRR306766, SRR306767, SRR306768, SRR306769, SRR306770, SRR306771, ERR2812391, ERR2812392, ERR2812393, ERR2812442, ERR2812443, ERR3417912, ERR3417983, ERR3418004, SRR649372, SRR943342, SRR943343, SRR649373, SRR649374, SRR943344, SRR943345, SRR943355, SRR943356, ERR2812397, ERR2812398, ERR2812399
Raw RNA-seq data from rat tissue samples: Brain, Liver, Muscle, Testis	Roller et al.[63]	ERR3417910, ERR3417913, ERR3417949, ERR3417917, ERR3417992, ERR3418010, ERR3417901, ERR3417903, ERR3417991, ERR3417900, ERR3417906, ERR3417950
Raw RNA-seq data from rabbit Human tissue samples: Brain, Liver, Muscle, Testis	Roller et al.[63]	ERR3417909, ERR3417957, ERR3417997, ERR3417919, ERR3417945, ERR3417948, ERR3417959, ERR3417978, ERR3417982, ERR3417971, ERR3417973,ERR3417980
Raw RNA-seq data from pig tissue samples: Brain, Liver, Muscle, Testis	Roller et al.[63]	ERR3417916, ERR3418014, ERR3418018, ERR3417918, ERR3417924, ERR3417937, ERR3417935, ERR3417965, ERR3417993, ERR3417904, ERR3417952, ERR3418012
Raw RNA-seq data from horse tissue samples: Brain, Liver, Muscle, Testis	Roller et al.[63]	ERR3418005, ERR3418007, ERR3418011, ERR3417940, ERR3417956, ERR3417976, ERR3417966, ERR3417981, ERR3417988, ERR3417911, ERR3417951, ERR3417969
Raw RNA-seq data from cat tissue samples: Brain, Liver, Muscle, Testis	Roller et al.[63]	ERR3417907, ERR3417925, ERR3417967, ERR3417927, ERR3417928, ERR3417934, ERR3417923, ERR3417932, ERR3417979, ERR3417926, ERR3417931, ERR3417933

(*Continued on next page*)

Continued		
REAGENT or RESOURCE	SOURCE	IDENTIFIER
Raw RNA-seq data from dog tissue samples: Brain, Liver, Muscle, Testis	Roller et al.[63]	ERR3417943, ERR3417972, ERR3417974, ERR3417942, ERR3417961, ERR3417977, ERR3417929, ERR3417936, ERR3417999, ERR3417958, ERR3417960, ERR3417985
Raw RNA-seq data from opposum tissue samples: Brain, Cerebellum, Heart, Kidney, Liver, Muscle, Ovary, Placenta, Testis	Wang et al.[61]; Brawand et al.[62]; Roller et al.[63]; Necsulea et al.[44]	ERR2812403, ERR2812404, ERR2812405, SRR306745, SRR306746, SRR306747, SRR306748, SRR306749, SRR306750, SRR306751, SRR306752, ERR2812409, ERR2812410, ERR2812411, ERR3417939, ERR3417954, ERR3417989, SRR649377, SRR943346, SRR649378, SRR943347, SRR943348, ERR2812415, ERR2812416, ERR2812417
Raw RNA-seq data from platypus tissue samples: Brain, Cerebellum, Heart, Kidney, Liver, Ovary, Testis	Wang et al.[61]; Brawand et al.[62]; Necsulea et al.[44]	ERR2812421, ERR2812422, ERR2812423, SRR306728, SRR306729, SRR306730, SRR306731, SRR306732, SRR306733, SRR306734, ERR2812427, ERR2812428, ERR2812429, SRR649382, SRR943349, SRR943350, ERR2812433, ERR2812434, ERR2812435
Raw RNA-seq data from chicken tissue samples: Brain, Cerebellum, Heart, Kidney, Liver, Ovary, Testis	Wang et al.[61]; Brawand et al.[62]; Necsulea et al.[44]	ERR2812331, ERR2812332, ERR2812333, SRR306712, SRR306713, SRR306714, SRR306715, SRR306716, SRR306717, ERR2812337, ERR2812338, ERR2812339, ERR2812438, ERR2812439, SRR649386, SRR649387, SRR649388, SRR943351, ERR2812343, ERR2812344, ERR2812345
Raw RNA-seq data from frog tissue samples: Brain, Heart, Kidney, Liver, Ovary, Testis	Necsulea et al.[44]	SRR649391, SRR649392, SRR649393, SRR649394, SRR649395, SRR649396, SRR649397, SRR649398, SRR649400, SRR943352, SRR649399, SRR943353
Software and algorithms		
LiftOver	UCSC Genome Browser	https://genome.ucsc.edu/cgi-bin/hgLiftOver
RaptorX	Wang et al.[64]	https://github.com/realbigws/Predict_Property
Phobius	Käll et al.[65]	https://phobius.sbc.su.se/
IUPRED	Dosztányi et al.[66]	https://iupred2a.elte.hu/
DeepLoc	Almagro Armenteros et al.[67]	https://services.healthtech.dtu.dk/service.php?DeepLoc-1.0
Bedtools	Quinlan et al.[68]	https://github.com/arq5x/bedtools2

(*Continued on next page*)

Continued		
REAGENT or RESOURCE	SOURCE	IDENTIFIER
CPAT	Wang et al.[48]	http://lilab.research.bcm.edu/
Codonw	CodonW[69]	http://codonw.sourceforge.net/
Cutadapt (v 3.7)	Martin[70]	https://cutadapt.readthedocs.io/en/stable/
STAR (v2.7.10a)	Dobin et al.[71]	https://github.com/alexdobin/STAR
StringTie	Pertea et al.[72]	https://ccb.jhu.edu/software/stringtie/
BLAST+ (v. 2)	Altschul et al.[73]	https://ftp.ncbi.nlm.nih.gov/blast/executables/blast+/LATEST/
MAFFT (v. 7.3)	Katoh et al.[74]	https://mafft.cbrc.jp/alignment/software/
Gotree (v. 0.4.1.a)	Lemoine et al.[75]	https://github.com/evolbioinfo/gotree
RAxML next generation (v.0.9.0)	Kozlov et al.[76]	https://github.com/amkozlov/raxml-ng
FastML (v.3.11)	Ashkenazy et al.[77]	http://fastml.tau.ac.il/source.php
TranslatorX	Abascal et al.[78]	http://translatorx.co.uk/
PhyloCSF	Lin et al.[46]	https://github.com/mlin/PhyloCSF
HyPhy (v.2.5.25)	HyPhy[79]	https://www.hyphy.org/
PAML	Yang et al.[80]	http://abacus.gene.ucl.ac.uk/software/paml.html#download

RESOURCE AVAILABILITY

Lead contact

Further information and requests for resources should be directed to and will be fulfilled by the lead contact, Nikolaos Vakirlis (vakirlisnikos@gmail.com).

Materials availability

This study did not generate new, unique reagents.

Data and code availability

- This paper analyzes existing, publicly available data. These accession numbers for the datasets are listed in the key resources table.
- This paper does not report original code
- Any additional information required to reanalyze the data reported in this paper is available from the lead contact upon request.

METHOD DETAILS

Data collection

Our dataset included ORFs that were identified as translated with high confidence by Chen et al.[42] based on analysis performed by the ORF-RATER program[81] (ORF-RATER score $\geq$ 0.8). Following the classification of Chen et al., we restricted our analysis to those ORFs located on either previously annotated non-coding transcripts ("new"), upstream of coding ORFs on coding transcripts ("upstream"), downstream of coding ORFs on coding transcripts ("downstream") or on transcripts lacking coding ORFs but which belong to a transcript family with a member annotated as coding ("new_iso"). We also required that ORFs be present in the comprehensive catalog established by Hon et al.[43] (FANTOM-CAT dataset). ORFs from the two studies were matched based on identical chromosomal coordinates, 100% sequence identity and identical length. Our final dataset consisted of 715 ORFs, located on 527 unique transcripts. Note that some of these ORFs overlap with others. Human genome version *hg19* coordinates were converted to *hg38* using the *liftover* tool in UCSC Genome Browser.

Various types of data were collected and generated for each ORF and its encoded protein. We considered whether the ORF was found to have significant fitness effects according to Chen et al.'s high-throughput CRISPR-Cas knockout screens in iPSC and K562 chronic myeloid leukemia cells. Phenotypic scores and classification (significant/not significant) were collected from the data of Chen et al. for each ORF in the two cell lines. Orthologous transcription, various coding signatures for ORFs and transcripts, expression data, cell type association, trait association, transcription properties for each transcript were obtained from the Data S1 and the raw data depository of Hon et al.[43] Protein secondary structure was predicted by RaptorX[64] using default parameters, transmembrane domains were predicted with Phobius,[65] disordered regions were predicted with IUPRED,[66] subcellular localization was predicted with DeepLoc[67] and percentage of aromatic and hydrophobic amino acids were calculated with codonw.[69] CPAT[48] was applied on the sequences of the ORFs to calculate the Hexamer and coding probability scores.

RNA-seq data processing and mapping

RNA-seq data for 17 species and up to 8 tissues, taken from 4 studies,[44,61–63] was obtained from SRA (Table S5). Reads were quality and adapter trimmed using TrimGalore (v 0.6.6) with cutadapt[70] (v 3.7) with a quality cutoff of 10. Reads for each sample were mapped to the relevant genome assembly (Table S1) using STAR[71] (v 2.7.10a) with default settings, except for muscle samples where –seedPerWindowNmax was set to 30. To ensure no influence of annotation differences on mapping, a 2-pass strategy was used in which the first mapping step was used to obtain splice junctions from the RNA-seq data and reads were then re-mapped using this additional information.

Improvement of transcript set and expression quantification with StringTie

Aligned reads were supplied to StringTie[72] along with reference annotations from UCSC for the relevant assembly. Multiple sets of parameters were used with varying stringencies; default, –conservative and parameters used in Wang et al. 2020.[61] We observed minimal differences and thus the latter set of parameters was used. Orthologous region overlap with assembled transcripts in each species was determined using *bedtools*[68] *intersect* and the maximum TPM value as calculated by StringTie for the overlapping transcripts was assigned as the expression value for each orthologous region of each ORF.

Inference of orthologous transcription based on reference transcriptomes

We initially inferred orthologous transcription by two means. First, we downloaded the NCBI RefSeq annotation GTF files for 92/99 vertebrate species for which it was available. Before it was possible to detect whether orthologous regions of ORFs were transcribed however, it was necessary to convert genomic coordinates from the assembly versions used in the 100-way alignments, to those used in RefSeq. To do this, we performed BLASTn[73] searches of the orthologous regions to their corresponding genome RefSeq assemblies using a cut-off of 97% identity. We were thus able to define the coordinates of each exon in the RefSeq version of the assemblies. We then verified that all updated coordinates produced in this manner were indeed as close as expected to the previous ones (i.e. we confirmed that no irrelevant matches were retrieved). We then checked, in each species, whether at least one exon of each orthologous region overlapped with an annotated transcript. An 80% overlapping cut-off was used. This gave us an initial pattern of presence and absence of transcription across the 92 species. Furthermore, we performed additional BLASTn searches of each human ORF to the entire vertebrate RefSeq transcript sequence database (downloaded March of 2021 from NCBI's ftp website, https://ftp.ncbi.nlm.nih.gov/refseq/release/vertebrate_mammalian/plus https://ftp.ncbi.nlm.nih.gov/refseq/release/vertebrate_other/) and considered the region as transcribed if it matched at the sequence level (regardless of genomic position) any transcript in one of the 99 species also present in the 100 vertebrate UCSC dataset, with at least 60% query coverage and at least 0.001 E-value. Note that only very rarely a presence was inferred in this manner that was not also retrieved based on annotation overlap. The union of all species with orthologous transcription based on the two approaches, across the 92 vertebrate species, was used to we define the initial timing of origination of the transcript as the most recent common ancestor of all the species for which a presence was inferred (Dollo parsimony). To do this, we used the 100-way vertebrate phylogenetic tree from the UCSC genome browser. Phylogenetic nodes were then manually matched to their official taxonomic names through TimeTree.org.[82]

Inference of orthologous transcription based on analysis of expression data

To improve our initial inference of orthologous transcription across vertebrates and our estimated transcriptional ages, we turned to the transcripts that we assembled using raw RNA-seq data (see previous Methods subsections). To infer an orthologous region of a human ORF as transcribed in a given species, we required non-zero overlap to a transcript with a minimum expression cut-off of 0.1 TPM. That cut-off was increased to 1 TPM in cases where the presence-absence pattern generated by the 0.1 TPM cut-off was highly sparse and incompatible with a phylogenetic origin of a consistently expressed transcript at the common ancestor of the species, which is what we are looking to identify here. These were defined as cases for which using a 1 TPM cut-off decreased by at least 50% the number of species descending from the common ancestor that had no inferred transcription. We additionally required that less than 80% of the species with inferred presence shared a common tissue with expression (that is, if the presence-absence pattern met the first criterion but the transcript was expressed in the same tissue in >80% of the species, the 0.1 TPM cut-off was maintained). The stricter cut-off was applied in 43/715 cases.

For each ORF, a final set of species with inferred orthologous transcription was constructed by adding any extra species recovered based on the analysis of raw expression data to the ones inferred using reference transcriptomes. A final timing of origination of transcript was then predicted for each ORF, as before. Overall, integrating the raw expression data led to a change of timing of origination for 169/715 cases. Out of these 169, 17 were found to have human-specific transcription (out of a total of 27 previously having human-specific transcription). Expression levels for these 27 ORFs and their orthologous regions are shown in Figure S7.

Validation of timing or origination of transcript using other transcriptome sources

Transcripts assembled in two previous studies[44,45] were used as a validation of our final inferred transcriptional ages. For Sarropoulos et al., exon coordinates of their transcripts were converted to the assemblies used in this study using the UCSC *liftOver* tool and overlaps were assigned using bedtools as described above. Any overlap was taken as presence of the transcript in that specific species. Timing of origination was then calculated as described above, by taking the most recent common ancestor in the UCSC vertebrate phylogenetic tree. For Necsulea et al., we inferred transcriptional ages in two different ways. First, as for Sarropoulos

et al., we used overlap to exons (taken from the ExonBlock* files, main dataset) and then inferred ages from most recent common ancestors. Second, we obtained correspondence from our human genes to the genes identified in that study using overlap of coordinates of entire transcripts. Note that this is extremely permissive as sometimes the specific ORFs we are interested in will not even be included in the exons of these transcripts, but we nevertheless included this approach to be maximally conservative. Once we had the correspondence of genes, we obtained the maximum inferred phylogenetic age of the family containing each gene, as calculated by Necsulea et al. for their main dataset.

Identification of orthologous genomic regions and inference of presence of ancestral ORFs

For each of the 715 human ORFs, we identified its orthologous region in 99 vertebrate genomes based on the UCSC Genome Browser 100-way, whole-genome alignments. The exact orthologous genomic regions corresponding to each exon of the human ORF were extracted using custom Python scripts. The regions corresponding to the different exons were then stitched together. For all ORFs, the orthologous region could be identified in a minimum of 4 other genomes. A multiple sequence alignment of each ORF together with its orthologous sequences was then performed using MAFFT.[74]

For each ORF, we first pruned the UCSC 100-way phylogenetic tree using the *gotree* tool[75] to keep only leaves corresponding to species present in the alignment of orthologous regions. A phylogenetic tree following the pruned tree's species topology was then constructed from the multiple alignment of each ORF and its orthologous sequences, using RAxML[76] (*raxml-ng –evaluate –msa ORF_alignment.fa –model GTR + G –tree pruned_ORF_tree.nwk*). Then, each multiple alignment and its corresponding tree were given as input to FastML,[77] to reconstruct the various ancestral sequences. The JC substitution matrix was used, and the ML method was used for reconstruction of indels. The marginal ancestral reconstructions were then parsed.

We examined the reconstructed sequences of the human ancestors. The origin of the human ORF was defined as the most ancient human ancestor in which at least 70% of the reconstructed ancestral sequence was an intact ORF (length of ancestral ORF/length of full ancestral sequence), i.e. any premature stop codons did not disrupt more than 30% of the length of the sequence (a 50% and an 80% cut-off was also used, see main text for details). The reading frame used was always on the forward strand, starting from the first position of the reconstructed sequence. If the ancestral sequence was longer than the human one, the length of the human sequence was used as the denominator of the ratio (length of ancestral ORF/length of human ORF). If the length of the reconstructed sequence was less than half the length of the human ORF, the ancestor was not taken into account, effectively considered as intact. Ancestral sequences were counted as intact ORFs regardless of whether an ATG start codon was present or not. We distinguished cases for which at least one disrupted (not intact) ancestor could be identified on a more ancient node than the one of predicted origin. For these cases, we were thus able to provide positive evidence of *de novo* formation: a disrupted ancestor that preceded the most ancient intact one. To be maximally conservative, we also conducted protein level similarity searches of all candidate ORFs, using BLASTp, against the annotated proteomes of all "outgroup" species, i.e. those diverged prior to the predicted node of origin of the ORF (proteomes downloaded from NCBI's RefSeq). Matches were deemed as significant if they had $<10^{-5}$ E-value, 40% identity and 50% query coverage. Based on these matches, we reassigned the node of origin to the most recent ancestor of the expanded set of species when necessary and removed de novo origin status. This was applied to 17 cases.

To detect possible inconsistencies with de novo origination, we performed a search for similarity to already annotated human protein sequences (Homo_sapiens.GRCh38.pep.all.fa file downloaded from ENSEMBL) using BLASTp with an E-value cut-off of 10^{-5}, 50% identity and 50% query coverage, providing as query our de novo originated ORFs. We recovered two matches. One of them was the protein itself (CATP00000191117.1 - > ENSP00000493702.1, 100% identity). We confirmed from ENSEMBL that the annotated gene (ENSG00000170846, which has only one protein associated) had the same predicted origin (Eutheria, from the Gene gain/loss tab) as the one we calculated, for both ORF and transcript. According to ENSEMBL, the gene also has two paralogues, which both originated at the root of Eutheria. The second match came from ORF CATP00001059838.1. This ORF again matches part of an ORF of its own gene (ENSG00000267360), but at 76% identity. The origin of the gene is more ancient (Boreoeutheria) than our predicted origin of the ORF (Simiiformes), but this is expected since this is an upstream ORF, and not the main coding ORF of the transcript. Overall, this search revealed no inconsistencies linked to human paralogues of our candidates.

The putative origin of each microprotein was defined as the earliest node on the phylogenetic tree on which both an ORF and transcription were present in a locus, unless we were able to detect a protein-coding signal using PhyloCSF (score >10), on the alignment containing only the species descending from the node of ORF origination. See the following subsection for details. Out of 312 microproteins for which ORF origination preceded transcription origination, only 33 satisfied this criterion, none of which had de novo status.

Functional signatures and statistics

We extracted all SNPs from dbSNP[51] within the coordinates of each of our ORFs that were not annotated as benign, using the following command, for each exon:

Esearch -db snp -query CHR_NO and (START_COORD:STOP_COORD) NOT "benign"[Clinical Ssgnificance])" | efetch -format json

Detailed information for each SNP was then retrieved from the SNP's page at dbSNP and ClinVar.

To calculate PhyloCSF[46] scores, we placed the human ORF sequence and orthologous sequences in species descending from the phylogenetic node of origin of the ORF in a FASTA file. We took the origin of the ORF and not the putative origin of the microprotein to minimize cases of origin age underestimation due to incomplete transcript annotation, as mentioned in the main text. We then

generated a codon-aware nucleotide alignment with the TranslatorX[78] tool, keeping the reading frame unchanged. PhyloCSF scores were then calculated based on these alignments, using the human sequence as reference and the –removeRefGaps option, searching only in the first reading frame and employing the ''vertebrates100'' model. PhyloCSF was applied by Chen et al. on alignments including sequences from 10 mammals spanning the Euarchontoglires, and by Hon et al. on alignments including 27 mammalian species. To identify selection/coding signatures on exemplar ORF CATP00001771233.1 we used two alignments: one containing all 47 orthologous sequences (not codon-aware) and one containing only the 11 primates (this alignment contained no change of frame). On both alignments, we run PhyloCSF as above but with the *-f 6* option to calculate a score for each frame, and the HyPhy program FEL[79] (default mode) and the PAML[80] program codeml (model = 0, nsites = 0) to estimate global dN/dS ratios, using the phylogenetic tree for the specific ORF reconstructed as described previously (the tree was pruned for use with the primates alignment). FEL and codeml were run independently on all 6 frames of the alignment, which we generated. Before each run, we removed alignment positions containing in-frame stop codons.

QUANTIFICATION AND STATISTICAL ANALYSIS

All statistics were done in R version 3.6.2. Plots were generated using ggplot2.[83] All statistical details including the type of statistical test performed and exact value of n (n always represents number of ORFs or microproteins) can be found in the Results and figure legends. Boxplots show median (horizontal line inside the box), first and third quartiles of data (lower and upper hinges) and values no further or lower than 1.5*distance between the first and third quartiles (upper and lower whisker). No methods were used to determine whether the data met assumptions of the statistical approaches.

Cell Reports, Volume *41*

Supplemental information

De novo birth of functional microproteins in the human lineage

Nikolaos Vakirlis, Zoe Vance, Kate M. Duggan, and Aoife McLysaght

Supplementary Figures

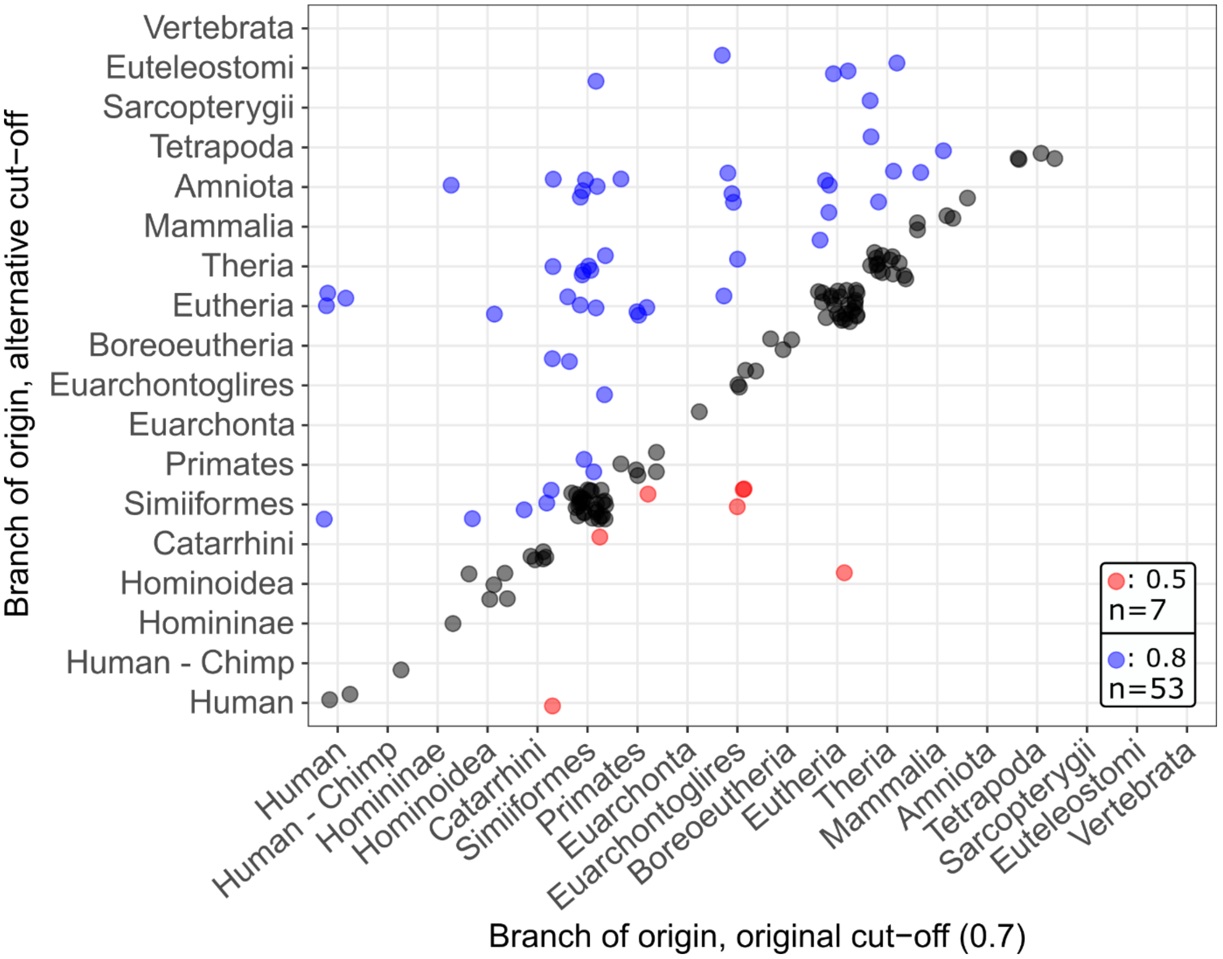

Figure S1: **Alternative intact length proportion cut-off comparison**. Effects of applying two alternative intact length proportion cut-offs, one stricter (red points) and one more relaxed (blue points) on predicted branch of ORF origin for 155 de novo originated ORFs. Related to Figure 1.

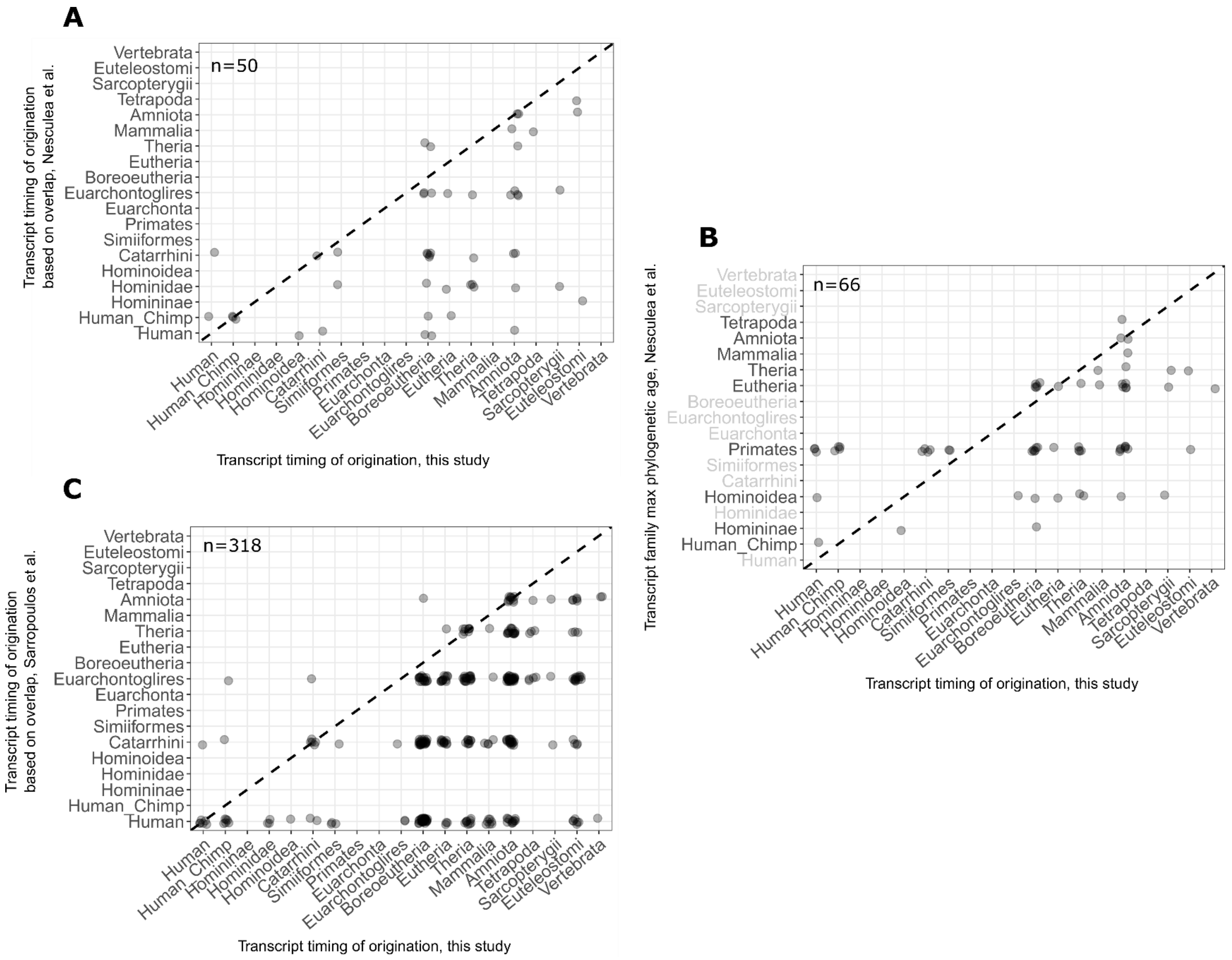

Figure S2: Transcript timing of origination comparison. A: Transcript timing of origination comparison using our data and using the assembled transcriptome data from Necsulea et al., relying on overlap of exonic regions and calculated by us (40/50 ages are older using our data, 4/50 ages are older using Nesculea et al. data, 6/50 the ages are the same; avg. time differential is 107.1my) B: Same comparison but using overlap of entire transcript coordinates of Necsulea et al. and taking the transcript family maximum age as calculated by Necsulea et al (numbers as in A, 42/66, 21/66, 3/66; time differential is 87.76my) C: In the same manner as in A, but using the data from Sarropoulos et al. (numbers as in A: 283/318, 6/318, 29/318; time differential is 134.8my) Related to Figure 1.

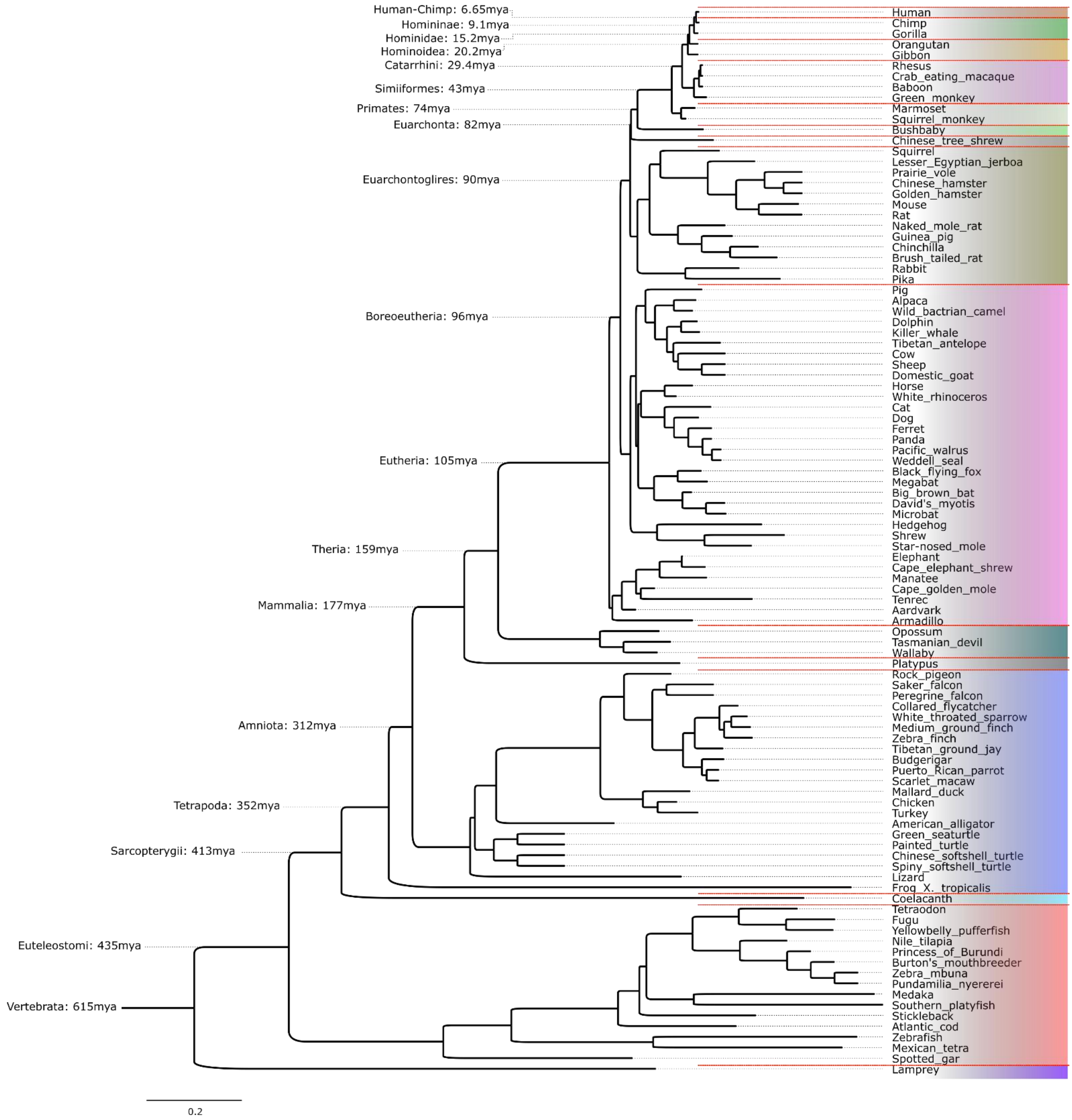

Figure S3: Phylogeny of species included in this study. UCSC Genome Browser 100-way phylogenetic tree with common species names, visualized using FigTree (https://github.com/rambaut/figtree) and annotated by us with the human ancestral branches and their ages as estimated by TimeTree (http://www.timetree.org/). Colors and red lines help distinguish the species descending from each ancestral human branch. Related to Figure 1.

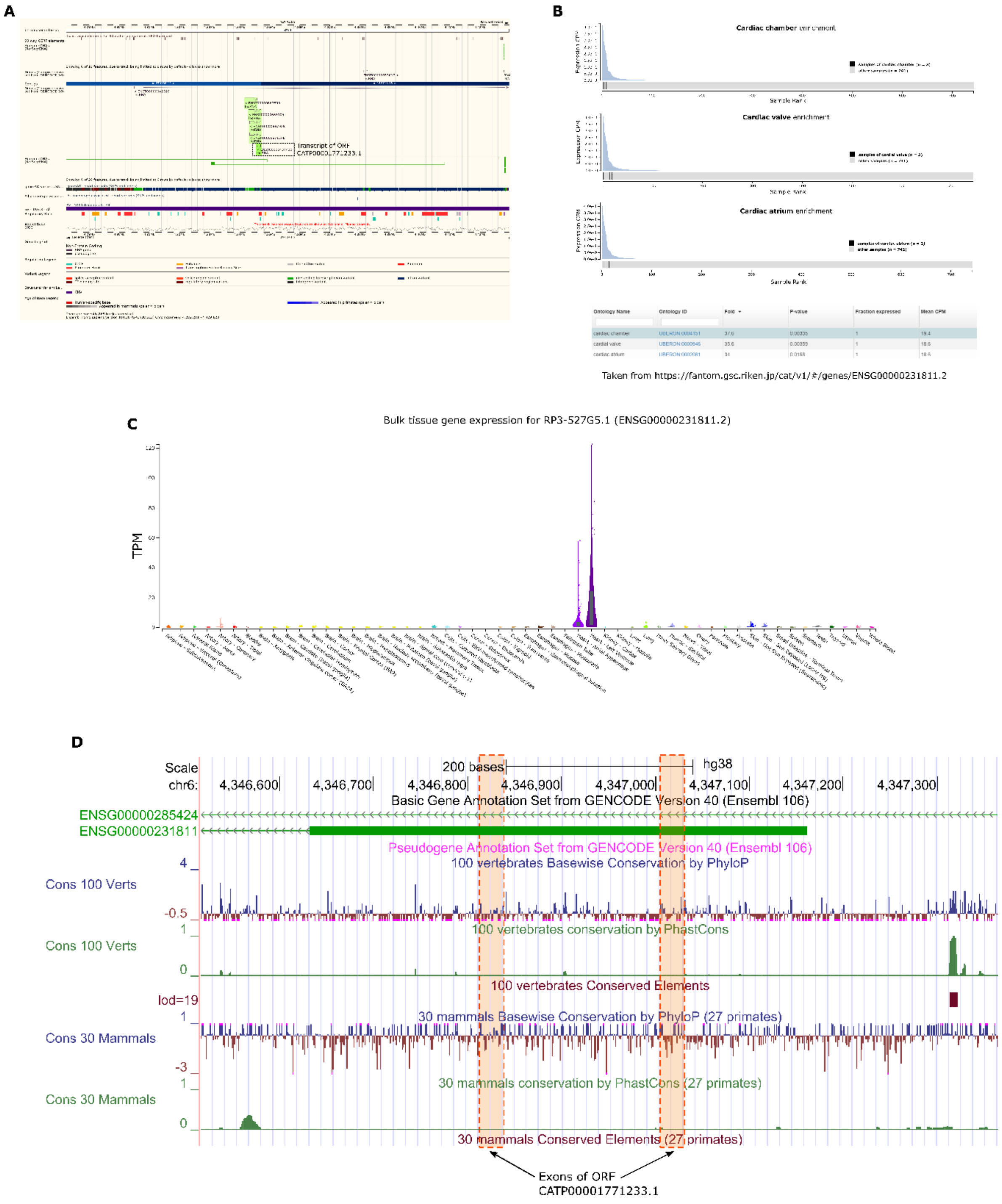

Figure S4: Genomic, expression and conservation data for ENSG00000231811.2. A: Genome browser view from ENSEMBL of the region of gene ENSG00000231811.2. The transcripts of the gene are highlighted in green. B: Sample ontology enrichment (three highest ranking ones) detected by Hon et al. for gene ENSG00000231811.2. Plots show the ranks of the samples for each ontology, table shows the relevant information. Taken from https://fantom.gsc.riken.jp/cat/v1/#/genes/ENSG00000231811.2 C: Expression plot taken from GTeX https://gtexportal.org/home/gene/RP3-527G5.1#geneExpression . D: UCSC

Genome Browser view of the region of the exemplar ORF; only conservation tracks are shown. Related to Figure 2.

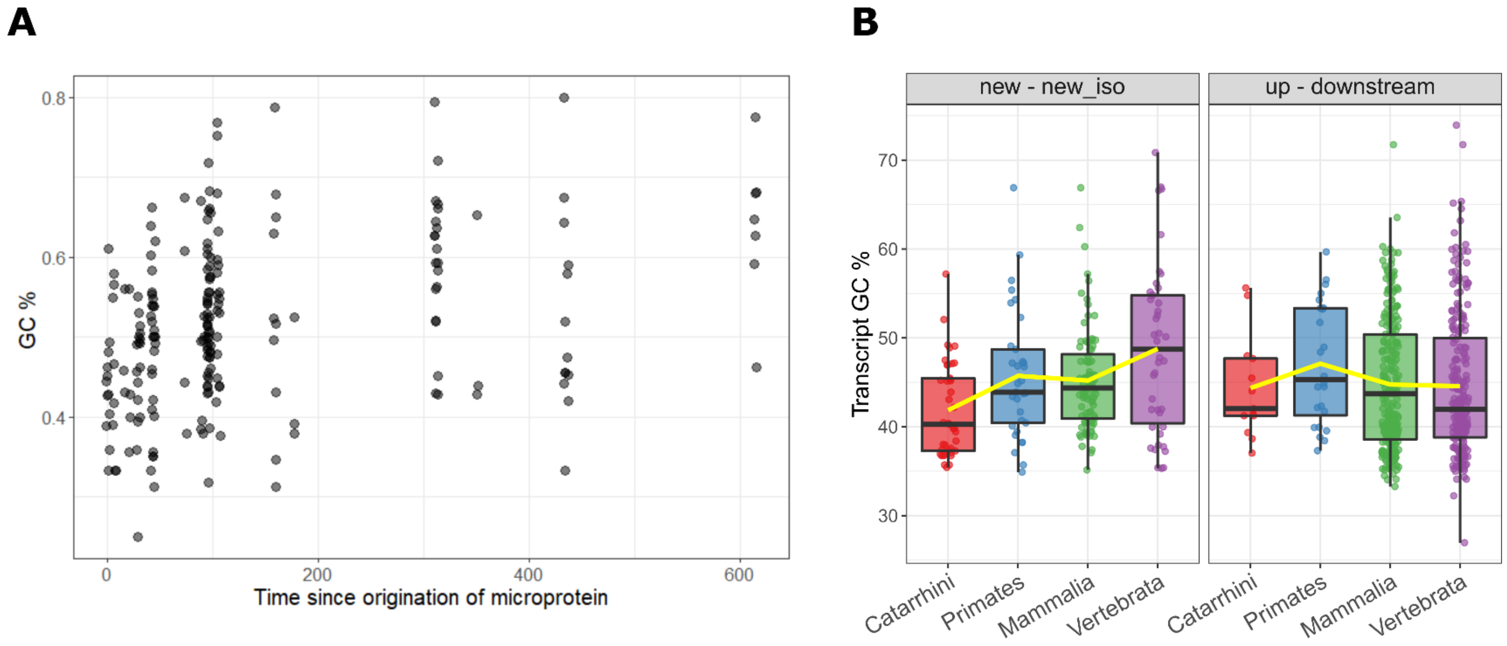

Figure S5: ORF and transcript GC% and timing of origination. A: Correlation of ORF GC% to time since putative origin of microprotein, for ORFs on new/new_iso transcripts (Spearman's Rho=0.36, P-value=5x10^{-8}). B: GC% of main transcripts for ORFs in the four broad groups of origin. Difference between Catarrhini and Vertebrata groups in the "new – new_iso" type is statistically significant (Wilcoxon test P-value=0.0004, means of 41.9 vs. 48.8). Contrary to ORF GC%, there is no statistically significant difference between those in the "new – new_iso" and those in the "up – downstream" types (P-value = 0.16). Related to Figure 3.

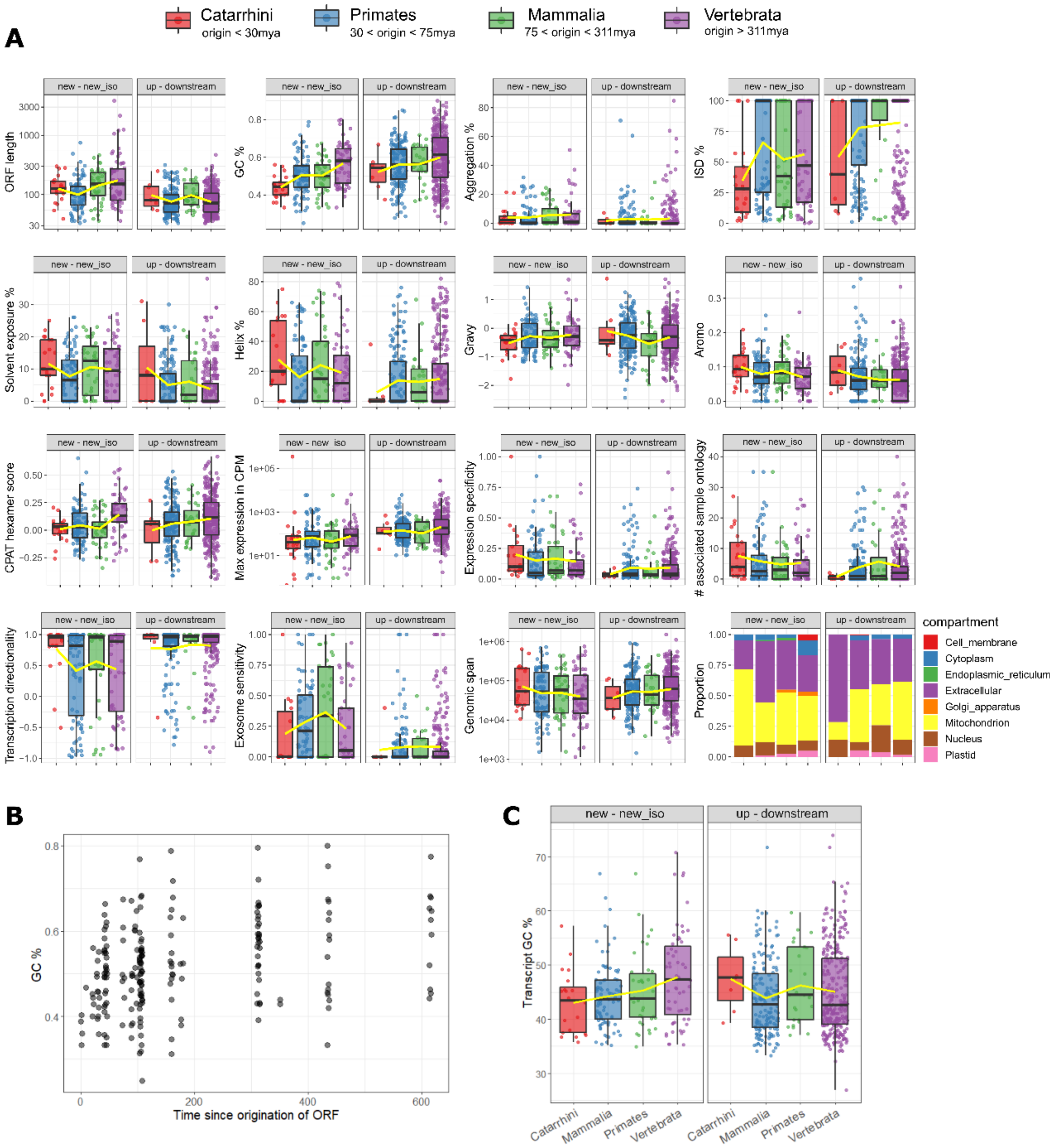

Figure S6: Distributions of various ORF, transcript and protein properties (ORF-based timing of origination definition). A: Distributions of various ORF, transcript and protein properties for all 715 microproteins, in four broad groups of putative origin age, defined using ORF timing or origination only, for all cases. Yellow line connects the averages across the groups. B: Correlation of ORF GC% to timing of origination of ORF, for ORFs on new/new_iso transcripts (Spearman's Rho=0.32, P-value=1.8x10^{-6}). C: GC% of main transcripts for ORFs in the four broad groups of origin, defined by ORF origin. Difference between Catarrhini and Vertebrata groups in the "new – new_iso" type is statistically significant (Wilcoxon test P-value=0.025, means of 43.1 vs. 47.8). Related to Figure 3.

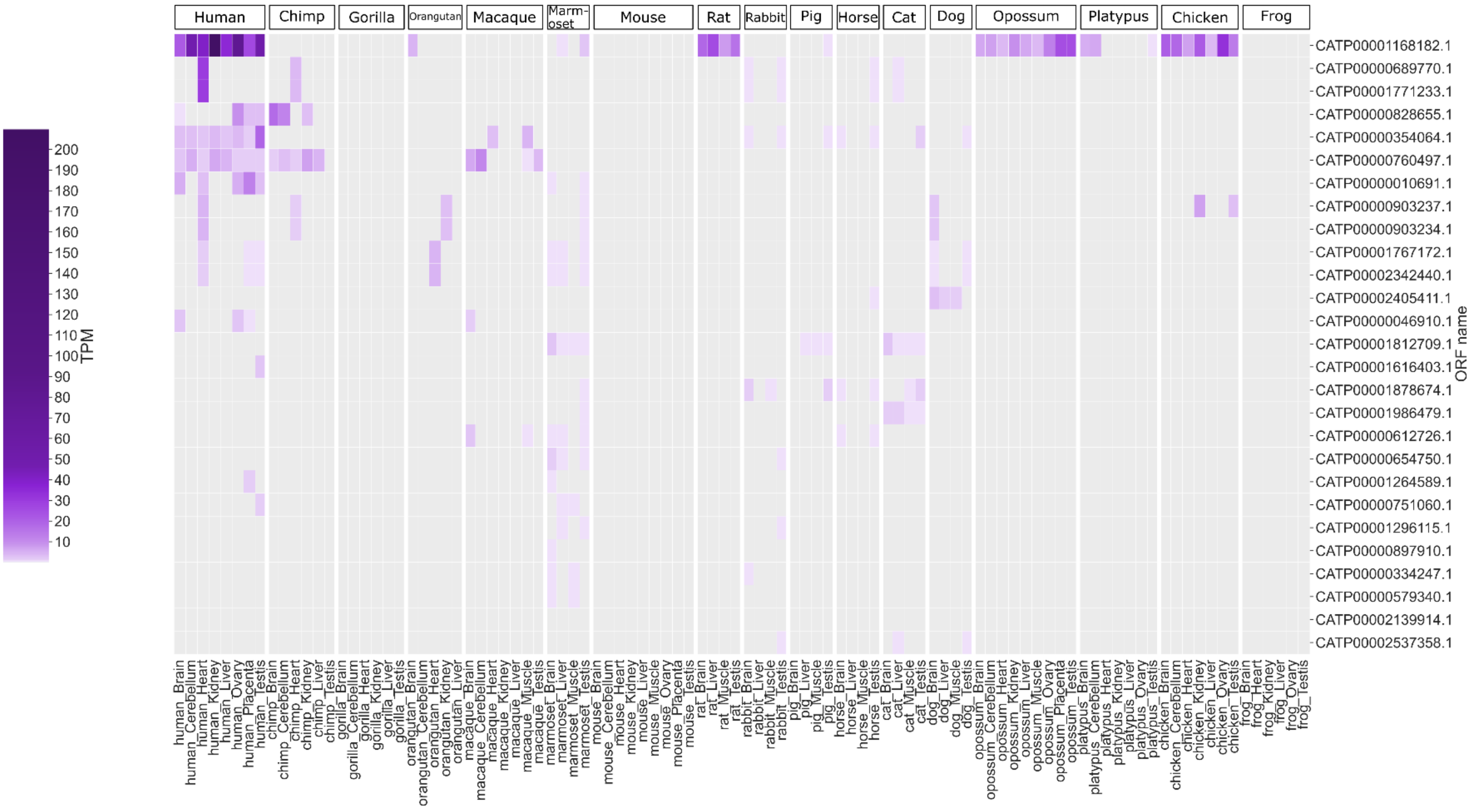

Figure S7: Expression levels of 27 ORFs initially predicted to be human-specific. Heatmap showing expression levels in TPM for each of the 27 ORFs initially predicted to be human-specific (based on analysis of reference transcriptomes) in the different tissue samples analyzed. 9/27 remain human-specific after filtering and integration of these data (see Methods). Related to Methods.
